# Changes in gut flora in patients with epilepsy: a systematic review and meta-analysis

**DOI:** 10.3389/fmicb.2024.1480022

**Published:** 2024-11-14

**Authors:** Xingyan He, Yuxin Zhang

**Affiliations:** ^1^Department of Pediatrics, Guangdong Provincial People's Hospital (Guangdong Academy of Medical Sciences), Southern Medical University, Guangzhou, Guangdong, China; ^2^Shantou University Medical College, Shantou, Guangdong, China

**Keywords:** epilepsy, intestinal flora, meta-analysis, gut-brain axis, gut microbiome

## Abstract

**Background:**

Epilepsy is a prevalent chronic neurological disorder that is strongly associated with a wide range of psychological, cognitive and social problems. It affects a significant proportion of the global population and has a number of complex etiologies. A growing body of research indicates that there is a strong association between epilepsy and the gut microbiota. Indeed, a substantial body of research has investigated the potential role of epilepsy in relation to the gut microbiota, examining alterations in the abundance, diversity, and relative abundance of the gut microbiota in patients with epilepsy.

**Methods:**

This study was conducted in accordance with the PRISMA guidelines and included multiple studies that met specific criteria. A keyword search was conducted in the following databases: PubMed, Embase, and Web of Science. The data extraction and quality assessment were conducted by two independent researchers. A systematic review and meta-analysis of the relationship between patients with epilepsy and gut flora was conducted using the R 4.3.4 software.

**Results:**

The results of the analyses indicated that the intestinal flora of patients with epilepsy did not differ significantly in alpha diversity compared to healthy controls. However, the relative abundance of specific flora, such as *Verrucomicrobia* and *Ackermannia* was significantly increased in patients, whereas Lactobacillus was significantly decreased.

**Conclusion:**

The relationship between epilepsy and gut flora is reciprocal. The present meta-analysis demonstrated that there were no statistically significant alterations in the overall characteristics of the intestinal flora of the patients. However, significant changes were observed in the relative abundance of certain phyla and genera. Consequently, it is hypothesized that epilepsy can cause changes in the relative abundance of specific flora in patients. Furthermore, in conjunction with previous studies, it is believed that changes in intestinal flora can also have an effect on seizures. For example, *Lactobacillus* may be a beneficial genus that potentially reduces seizures. Conversely, the effect of Akkermansia is two-sided.

## 1 Background

Epilepsy is a chronic and devastating neurological disorder characterized by seizures (Thijs et al., 2019). Epilepsy is often associated with psychological, cognitive, and social consequences (Fisher et al., 2014). Approximately 50 million people and 0.5–1% of children worldwide suffer from epilepsy (Aaberg et al., 2017). The incidence of epilepsy is highest in infancy, with approximately 0.67% of children diagnosed with epilepsy in the first 10 years of life (Elliott et al., 2019). The causes of epilepsy can be broadly categorized into six groups: genetic, structural, metabolic, immunologic, immune, infectious, and unknown factors (Scheffer et al., 2017; Fisher et al., 2017; Fisher et al., 2017).

Most people with epilepsy live in areas with poor sanitation (World Health Organization, 2024), and environmental factors have been shown to have a significant impact on the composition of the gut microbiota (Gacesa et al., 2022). Prior research has demonstrated that the utilization of antibiotics can potentially exacerbate seizures (Sutter et al., 2015). Furthermore, a high-fat ketogenic diet has been demonstrated to be an efficacious treatment for medically refractory epilepsy (Kwan and Brodie, 2000; Newman, 2000). Additionally, studies have indicated that the gut flora is a mediator of the antiepileptic effects of the ketogenic diet. In conclusion, the role of gut flora in the development or remission of epilepsy is significant. The gut-brain axis has been demonstrated to perceive and respond to alterations in the dynamic ecosystem by translating chemical signals from the environment and gut microbes into neural signals, thereby indicating a pivotal function for the gut microbiota in epileptogenesis (Tremlett et al., 2017).

To date, a number of systematic evaluations have demonstrated an association between epilepsy and the gut microbiota. This systematic review and meta-analysis aimed to examine the relationship between epilepsy and gut flora, with the objective of synthesizing and critically evaluating the available evidence supporting the interaction between gut microbiota and epilepsy, and elucidating potential interventions.

## 2 Methods

### 2.1 Search strategy

This systematic review and meta-analysis is conducted in accordance with the Preferred Reporting Items for Systematic Reviews and Meta-Analyses (PRISMA) guidelines (Page et al., 2021).

**Figure 1:**
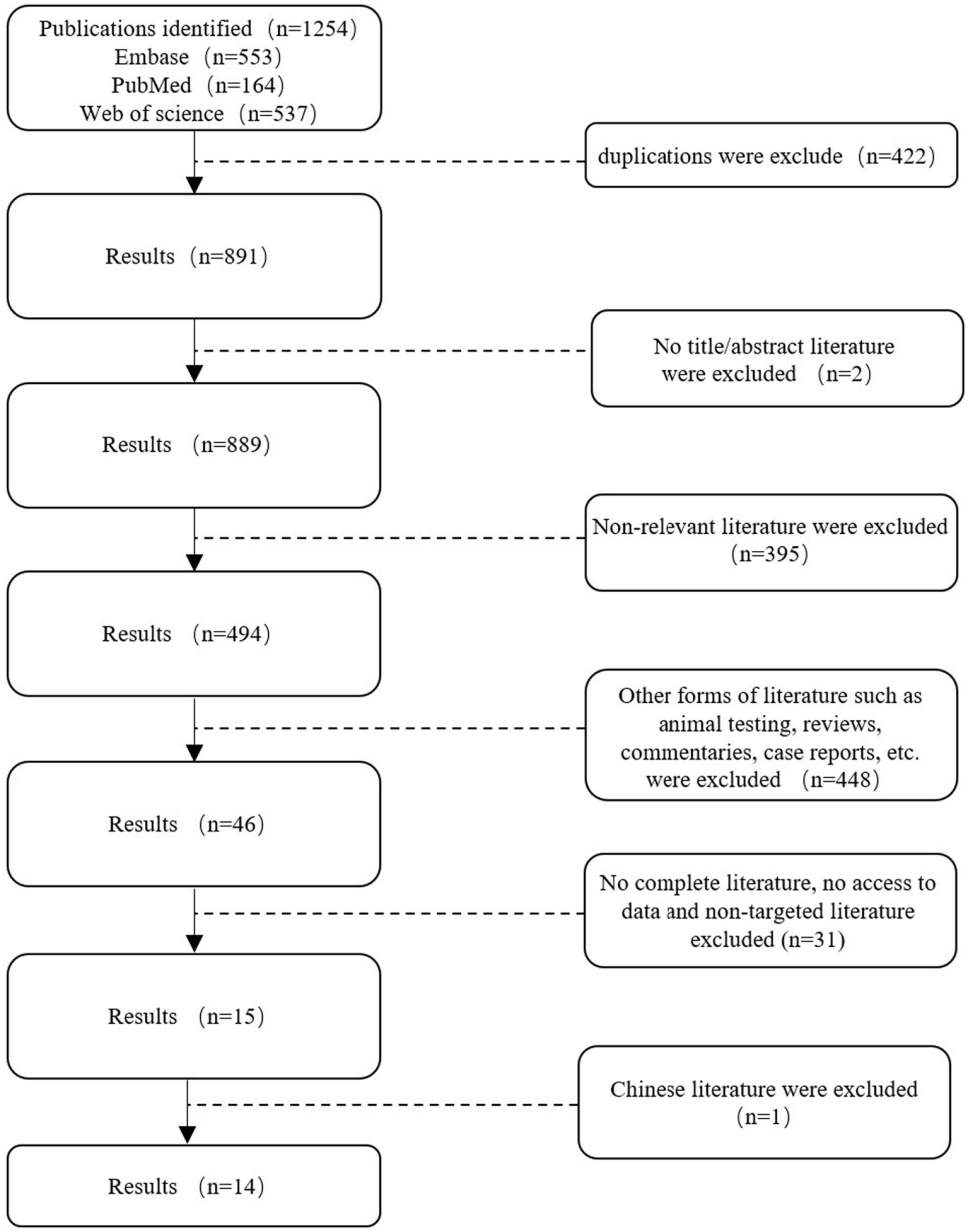
Search flowchart.

The following keywords were used for the analysis: epilepsy and intestinal flora. The keywords are presented in the following format: Medical Subject Headings + free word. A comprehensive search was conducted in PubMed, Embase, and Web of Science using the keywords “intestinal flora” and “epilepsy” to identify studies involving valuable epilepsy and intestinal flora analyses from database creation to 9 June 2024 (the search strategy refers to Table 1). The search language was English (Figure 1).

**Table 1:** The search strategy.

Embase			
No.	Query	Results	Date
#14	#12 AND #13	553	07-Jun-24
#13	#9 OR #10 OR #11	511865	07-Jun-24
#12	#7 OR #8	132153	07-Jun-24
#11	#5 OR #6	55652	07-Jun-24
#10	#3 OR #4	318515	07-Jun-24
#9	#1 OR #2	356264	07-Jun-24
#8	'alimentary canal flora' OR 'alimentary tract flora' OR 'bowel flora' OR 'bowel microbiota' OR 'digestive canal flora' OR 'digestive tract flora' OR 'enteric flora' OR 'enteric microbiota' OR 'flora, intestine' OR 'gastro intestinal flora' OR 'gastrointestinal canal flora' OR 'gastrointestinal flora' OR 'gastrointestinal microbiome' OR 'gastrointestinal microbiota' OR 'gastrointestinal tract flora' OR 'gastrointestine flora' OR 'gastrointestine tract flora' OR 'gut bacteria' OR 'gut microbiota' OR 'intestinal bacteria' OR 'intestinal bacterial flora' OR 'intestinal bacterium' OR 'intestinal canal flora' OR 'intestinal flora' OR 'intestinal microbe' OR 'intestinal microbes' OR 'intestinal microbiota' OR 'intestinal microflora' OR 'intestinal microorganism' OR 'intestinal tract flora' OR 'intestine bacteria' OR 'intestine bacteria change' OR 'intestine bacterial flora' OR 'intestine bacterium' OR 'intestine microbial flora' OR 'intestine microflora' OR 'intestine flora'	130315	07-Jun-24
#7	'intestine flora'/exp	111879	07-Jun-24
#6	'convulsion susceptibility' OR 'convulsions' OR 'convulsive action' OR 'convulsive disorder' OR 'convulsive fit' OR 'convulsive motor seizure' OR 'convulsive reaction' OR 'electroconvulsion' OR 'convulsion'	55126	07-Jun-24
#5	'convulsion'/exp	41516	07-Jun-24
#4	'seizure activities' OR 'seizure activity' OR 'seizure disorder' OR 'seizures' OR 'seizure'	312638	07-Jun-24
#3	'seizure'/exp	240303	07-Jun-24
#2	'acute epilepsy' OR 'attack, epileptic' OR 'cerebral seizure, epileptic' OR 'chronic epilepsy' OR 'comitial disease' OR 'convulsion, epileptic' OR 'convulsive epilepsy' OR 'epilepsia' OR 'epilepsy, convulsive' OR 'epileptic' OR 'epileptic attack' OR 'epileptic convulsion' OR 'epileptic disorder' OR 'epileptic fit' OR 'epileptic insult' OR 'epileptic seizure' OR 'epileptic seizure, cerebral' OR 'epileptic syndrome' OR 'epileptic syndromes' OR 'falling sickness' OR 'fit, epileptic' OR 'seizure, epileptic' OR 'sickness, falling' OR 'tardy epilepsy' OR 'epilepsy'	319581	07-Jun-24
#1	'epilepsy'/exp	307005	07-Jun-24

Pubmed			
No.	Query	Results	Time
8	(((((((((((((((((((((((((((((((((((((("Gastrointestinal Microbiome"[Mesh]) OR (Gastrointestinal Microbiomes[Title/Abstract])) OR (Microbiome, Gastrointestinal[Title/Abstract])) OR (Gastrointestinal Microbial Community[Title/Abstract])) OR (Gastrointestinal Microbial Communities[Title/Abstract])) OR (Microbial Community, Gastrointestinal[Title/Abstract])) OR (Gut Microbiome[Title/Abstract])) OR (Gut Microbiomes[Title/Abstract])) OR (Microbiome, Gut[Title/Abstract])) OR (Gut Microflora[Title/Abstract])) OR (Microflora, Gut[Title/Abstract])) OR (Gastrointestinal Microflora[Title/Abstract])) OR (Microflora, Gastrointestinal[Title/Abstract])) OR (Gastrointestinal Flora[Title/Abstract])) OR (Flora, Gastrointestinal[Title/Abstract])) OR (Gut Flora[Title/Abstract])) OR (Flora, Gut[Title/Abstract])) OR (Gastrointestinal Microbiota[Title/Abstract])) OR (Gastrointestinal Microbiotas[Title/Abstract])) OR (Microbiota, Gastrointestinal[Title/Abstract])) OR (Gut Microbiota[Title/Abstract])) OR (Gut Microbiotas[Title/Abstract])) OR (Microbiota, Gut[Title/Abstract])) OR (Intestinal Microbiome[Title/Abstract])) OR (Intestinal Microbiomes[Title/Abstract])) OR (Microbiome, Intestinal[Title/Abstract])) OR (Intestinal Flora[Title/Abstract])) OR (Flora, Intestinal[Title/Abstract])) OR (Intestinal Microbiota[Title/Abstract])) OR (Intestinal Microbiotas[Title/Abstract])) OR (Microbiota, Intestinal[Title/Abstract])) OR (Intestinal Microflora[Title/Abstract])) OR (Microflora, Intestinal[Title/Abstract])) OR (Enteric Bacteria[Title/Abstract])) OR (Bacteria, Enteric[Title/Abstract])) OR (Gastric Microbiome[Title/Abstract])) OR (Gastric Microbiomes[Title/Abstract])) OR (Microbiome, Gastric[Title/Abstract])) AND ((((((((((((("Epilepsy"[Mesh]) OR (Epilepsies[Title/Abstract])) OR (Seizure Disorder[Title/Abstract])) OR (Seizure Disorders[Title/Abstract])) OR (Epilepsy, Cryptogenic[Title/Abstract])) OR (Cryptogenic Epilepsies[Title/Abstract])) OR (Cryptogenic Epilepsy[Title/Abstract])) OR (Epilepsies, Cryptogenic[Title/Abstract])) OR (Aura[Title/Abstract])) OR (Auras[Title/Abstract])) OR (Awakening Epilepsy[Title/Abstract])) OR (Epilepsy, Awakening[Title/Abstract])) OR (((((((((((((((((((((((((((((((((((((((((((((((((((((((((((((((((((((((((((((((((((((((((((((((((((((((((((((((((((((((((((((((("Seizures"[Mesh]) OR (Seizure[Title/Abstract])) OR (Jacksonian Seizure[Title/Abstract])) OR (Seizure, Jacksonian[Title/Abstract])) OR (Single Seizure[Title/Abstract])) OR (Seizure, Single[Title/Abstract])) OR (Single Seizures[Title/Abstract])) OR (Atonic Absence Seizures[Title/Abstract])) OR (Atonic Absence Seizure[Title/Abstract])) OR (Absence Seizure, Atonic[Title/Abstract])) OR (Absence Seizures, Atonic[Title/Abstract])) OR (Seizure, Atonic Absence[Title/Abstract])) OR (Seizures, Focal[Title/Abstract])) OR (Focal Seizure[Title/Abstract])) OR (Focal Seizures[Title/Abstract])) OR (Seizure, Focal[Title/Abstract])) OR (Partial Seizures[Title/Abstract])) OR (Partial Seizure[Title/Abstract])) OR (Seizure, Partial[Title/Abstract])) OR (Seizures, Generalized[Title/Abstract])) OR (Generalized Seizure[Title/Abstract])) OR (Generalized Seizures[Title/Abstract])) OR (Seizure, Generalized[Title/Abstract])) OR (Seizures, Sensory[Title/Abstract])) OR (Seizure, Sensory[Title/Abstract])) OR (Sensory Seizure[Title/Abstract])) OR (Sensory Seizures[Title/Abstract])) OR (Seizures, Auditory[Title/Abstract])) OR (Auditory Seizure[Title/Abstract])) OR (Auditory Seizures[Title/Abstract])) OR (Seizure, Auditory[Title/Abstract])) OR (Convulsive Seizures[Title/Abstract])) OR (Seizure, Convulsive[Title/Abstract])) OR (Seizures, Convulsive[Title/Abstract])) OR (Seizures, Motor[Title/Abstract])) OR (Motor Seizure[Title/Abstract])) OR (Motor Seizures[Title/Abstract])) OR (Seizure, Motor[Title/Abstract])) OR (Convulsive Seizure[Title/Abstract])) OR (Seizures, Gustatory[Title/Abstract])) OR (Gustatory Seizure[Title/Abstract])) OR (Gustatory Seizures[Title/Abstract])) OR (Seizure, Gustatory[Title/Abstract])) OR (Seizures, Olfactory[Title/Abstract])) OR (Olfactory Seizure[Title/Abstract])) OR (Olfactory Seizures[Title/Abstract])) OR (Seizure, Olfactory[Title/Abstract])) OR (Seizures, Somatosensory[Title/Abstract])) OR (Seizure, Somatosensory[Title/Abstract])) OR (Somatosensory Seizure[Title/Abstract])) OR (Somatosensory Seizures[Title/Abstract])) OR (Seizures, Vertiginous[Title/Abstract])) OR (Seizure, Vertiginous[Title/Abstract])) OR (Vertiginous Seizure[Title/Abstract])) OR (Vertiginous Seizures[Title/Abstract])) OR (Seizures, Vestibular[Title/Abstract])) OR (Seizure, Vestibular[Title/Abstract])) OR (Vestibular Seizure[Title/Abstract])) OR (Vestibular Seizures[Title/Abstract])) OR (Seizures, Visual[Title/Abstract])) OR (Seizure, Visual[Title/Abstract])) OR (Visual Seizure[Title/Abstract])) OR (Visual Seizures[Title/Abstract])) OR (Convulsion, Non-Epileptic[Title/Abstract])) OR (Convulsion, Non Epileptic[Title/Abstract])) OR (Convulsions, Non-Epileptic[Title/Abstract])) OR (Non-Epileptic Convulsion[Title/Abstract])) OR (Non-Epileptic Convulsions[Title/Abstract])) OR (Nonepileptic Seizures[Title/Abstract])) OR (Non-Epileptic Seizures[Title/Abstract])) OR (Non Epileptic Seizures[Title/Abstract])) OR (Nonepileptic Seizure[Title/Abstract])) OR (Seizure, Nonepileptic[Title/Abstract])) OR (Seizures, Nonepileptic[Title/Abstract])) OR (Non-Epileptic Seizure[Title/Abstract])) OR (Non Epileptic Seizure[Title/Abstract])) OR (Seizure, Non-Epileptic[Title/Abstract])) OR (Complex Partial Seizures[Title/Abstract])) OR (Complex Partial Seizure[Title/Abstract])) OR (Partial Seizure, Complex[Title/Abstract])) OR (Partial Seizures, Complex[Title/Abstract])) OR (Seizure, Complex Partial[Title/Abstract])) OR (Epileptic Seizures[Title/Abstract])) OR (Seizures, Epileptic[Title/Abstract])) OR (Epileptic Seizure[Title/Abstract])) OR (Seizure, Epileptic[Title/Abstract])) OR (Generalized Absence Seizures[Title/Abstract])) OR (Generalized Absence Seizure[Title/Abstract])) OR (Generalized Absence Seizure[Title/Abstract])) OR (Absence Seizures, Generalized[Title/Abstract])) OR (Seizure, Generalized Absence[Title/Abstract])) OR (Tonic-Clonic Seizures[Title/Abstract])) OR (Seizures, Tonic-Clonic[Title/Abstract])) OR (Generalized Tonic-Clonic Seizures[Title/Abstract])) OR (Generalized Tonic-Clonic Seizure[Title/Abstract])) OR (Generalized Tonic Clonic Seizures[Title/Abstract])) OR (Seizure, Generalized Tonic-Clonic[Title/Abstract])) OR (Seizures, Generalized Tonic-Clonic[Title/Abstract])) OR (Tonic-Clonic Seizure, Generalized[Title/Abstract])) OR (Tonic-Clonic Seizures, Generalized[Title/Abstract])) OR (Tonic Clonic Seizure[Title/Abstract])) OR (Clonic Seizures, Tonic[Title/Abstract])) OR (Clonic Seizure, Tonic[Title/Abstract])) OR (Seizure, Tonic Clonic[Title/Abstract])) OR (Tonic Clonic Seizures[Title/Abstract])) OR (Tonic-Clonic Seizure[Title/Abstract])) OR (Seizure, Tonic-Clonic[Title/Abstract])) OR (Myoclonic Seizures[Title/Abstract])) OR (Myoclonic Seizure[Title/Abstract])) OR (Seizure, Myoclonic[Title/Abstract])) OR (Clonic Seizures[Title/Abstract])) OR (Seizures, Clonic[Title/Abstract])) OR (Clonic Seizure[Title/Abstract])) OR (Seizure, Clonic[Title/Abstract])) OR (Tonic Seizures[Title/Abstract])) OR (Seizures, Tonic[Title/Abstract])) OR (Tonic Seizure[Title/Abstract])) OR (Seizure, Tonic[Title/Abstract])) OR (Atonic Seizures[Title/Abstract])) OR (Atonic Seizure[Title/Abstract])) OR (Seizure, Atonic[Title/Abstract])) OR (Convulsions[Title/Abstract])) OR (Convulsion[Title/Abstract])) OR (Absence Seizures[Title/Abstract])) OR (Petit Mal Convulsion[Title/Abstract])) OR (Convulsion, Petit Mal[Title/Abstract])) OR (Absence Seizure[Title/Abstract])) OR (Seizure, Absence[Title/Abstract])))	164	21:48:26
7	((((((((((((((((((((((((((((((((((((("Gastrointestinal Microbiome"[Mesh]) OR (Gastrointestinal Microbiomes[Title/Abstract])) OR (Microbiome, Gastrointestinal[Title/Abstract])) OR (Gastrointestinal Microbial Community[Title/Abstract])) OR (Gastrointestinal Microbial Communities[Title/Abstract])) OR (Microbial Community, Gastrointestinal[Title/Abstract])) OR (Gut Microbiome[Title/Abstract])) OR (Gut Microbiomes[Title/Abstract])) OR (Microbiome, Gut[Title/Abstract])) OR (Gut Microflora[Title/Abstract])) OR (Microflora, Gut[Title/Abstract])) OR (Gastrointestinal Microflora[Title/Abstract])) OR (Microflora, Gastrointestinal[Title/Abstract])) OR (Gastrointestinal Flora[Title/Abstract])) OR (Flora, Gastrointestinal[Title/Abstract])) OR (Gut Flora[Title/Abstract])) OR (Flora, Gut[Title/Abstract])) OR (Gastrointestinal Microbiota[Title/Abstract])) OR (Gastrointestinal Microbiotas[Title/Abstract])) OR (Microbiota, Gastrointestinal[Title/Abstract])) OR (Gut Microbiota[Title/Abstract])) OR (Gut Microbiotas[Title/Abstract])) OR (Microbiota, Gut[Title/Abstract])) OR (Intestinal Microbiome[Title/Abstract])) OR (Intestinal Microbiomes[Title/Abstract])) OR (Microbiome, Intestinal[Title/Abstract])) OR (Intestinal Flora[Title/Abstract])) OR (Flora, Intestinal[Title/Abstract])) OR (Intestinal Microbiota[Title/Abstract])) OR (Intestinal Microbiotas[Title/Abstract])) OR (Microbiota, Intestinal[Title/Abstract])) OR (Intestinal Microflora[Title/Abstract])) OR (Microflora, Intestinal[Title/Abstract])) OR (Enteric Bacteria[Title/Abstract])) OR (Bacteria, Enteric[Title/Abstract])) OR (Gastric Microbiome[Title/Abstract])) OR (Gastric Microbiomes[Title/Abstract])) OR (Microbiome, Gastric[Title/Abstract])	98,850	21:48:05
6	"Gastrointestinal Microbiome"[Mesh]	44,218	21:42:42
5	(((((((((((("Epilepsy"[Mesh]) OR (Epilepsies[Title/Abstract])) OR (Seizure Disorder[Title/Abstract])) OR (Seizure Disorders[Title/Abstract])) OR (Epilepsy, Cryptogenic[Title/Abstract])) OR (Cryptogenic Epilepsies[Title/Abstract])) OR (Cryptogenic Epilepsy[Title/Abstract])) OR (Epilepsies, Cryptogenic[Title/Abstract])) OR (Aura[Title/Abstract])) OR (Auras[Title/Abstract])) OR (Awakening Epilepsy[Title/Abstract])) OR (Epilepsy, Awakening[Title/Abstract])) OR (((((((((((((((((((((((((((((((((((((((((((((((((((((((((((((((((((((((((((((((((((((((((((((((((((((((((((((((((((((((((((((((("Seizures"[Mesh]) OR (Seizure[Title/Abstract])) OR (Jacksonian Seizure[Title/Abstract])) OR (Seizure, Jacksonian[Title/Abstract])) OR (Single Seizure[Title/Abstract])) OR (Seizure, Single[Title/Abstract])) OR (Single Seizures[Title/Abstract])) OR (Atonic Absence Seizures[Title/Abstract])) OR (Atonic Absence Seizure[Title/Abstract])) OR (Absence Seizure, Atonic[Title/Abstract])) OR (Absence Seizures, Atonic[Title/Abstract])) OR (Seizure, Atonic Absence[Title/Abstract])) OR (Seizures, Focal[Title/Abstract])) OR (Focal Seizure[Title/Abstract])) OR (Focal Seizures[Title/Abstract])) OR (Seizure, Focal[Title/Abstract])) OR (Partial Seizures[Title/Abstract])) OR (Partial Seizure[Title/Abstract])) OR (Seizure, Partial[Title/Abstract])) OR (Seizures, Generalized[Title/Abstract])) OR (Generalized Seizure[Title/Abstract])) OR (Generalized Seizures[Title/Abstract])) OR (Seizure, Generalized[Title/Abstract])) OR (Seizures, Sensory[Title/Abstract])) OR (Seizure, Sensory[Title/Abstract])) OR (Sensory Seizure[Title/Abstract])) OR (Sensory Seizures[Title/Abstract])) OR (Seizures, Auditory[Title/Abstract])) OR (Auditory Seizure[Title/Abstract])) OR (Auditory Seizures[Title/Abstract])) OR (Seizure, Auditory[Title/Abstract])) OR (Convulsive Seizures[Title/Abstract])) OR (Seizure, Convulsive[Title/Abstract])) OR (Seizures, Convulsive[Title/Abstract])) OR (Seizures, Motor[Title/Abstract])) OR (Motor Seizure[Title/Abstract])) OR (Motor Seizures[Title/Abstract])) OR (Seizure, Motor[Title/Abstract])) OR (Convulsive Seizure[Title/Abstract])) OR (Seizures, Gustatory[Title/Abstract])) OR (Gustatory Seizure[Title/Abstract])) OR (Gustatory Seizures[Title/Abstract])) OR (Seizure, Gustatory[Title/Abstract])) OR (Seizures, Olfactory[Title/Abstract])) OR (Olfactory Seizure[Title/Abstract])) OR (Olfactory Seizures[Title/Abstract])) OR (Seizure, Olfactory[Title/Abstract])) OR (Seizures, Somatosensory[Title/Abstract])) OR (Seizure, Somatosensory[Title/Abstract])) OR (Somatosensory Seizure[Title/Abstract])) OR (Somatosensory Seizures[Title/Abstract])) OR (Seizures, Vertiginous[Title/Abstract])) OR (Seizure, Vertiginous[Title/Abstract])) OR (Vertiginous Seizure[Title/Abstract])) OR (Vertiginous Seizures[Title/Abstract])) OR (Seizures, Vestibular[Title/Abstract])) OR (Seizure, Vestibular[Title/Abstract])) OR (Vestibular Seizure[Title/Abstract])) OR (Vestibular Seizures[Title/Abstract])) OR (Seizures, Visual[Title/Abstract])) OR (Seizure, Visual[Title/Abstract])) OR (Visual Seizure[Title/Abstract])) OR (Visual Seizures[Title/Abstract])) OR (Convulsion, Non-Epileptic[Title/Abstract])) OR (Convulsion, Non Epileptic[Title/Abstract])) OR (Convulsions, Non-Epileptic[Title/Abstract])) OR (Non-Epileptic Convulsion[Title/Abstract])) OR (Non-Epileptic Convulsions[Title/Abstract])) OR (Nonepileptic Seizures[Title/Abstract])) OR (Non-Epileptic Seizures[Title/Abstract])) OR (Non Epileptic Seizures[Title/Abstract])) OR (Nonepileptic Seizure[Title/Abstract])) OR (Seizure, Nonepileptic[Title/Abstract])) OR (Seizures, Nonepileptic[Title/Abstract])) OR (Non-Epileptic Seizure[Title/Abstract])) OR (Non Epileptic Seizure[Title/Abstract])) OR (Seizure, Non-Epileptic[Title/Abstract])) OR (Complex Partial Seizures[Title/Abstract])) OR (Complex Partial Seizure[Title/Abstract])) OR (Partial Seizure, Complex[Title/Abstract])) OR (Partial Seizures, Complex[Title/Abstract])) OR (Seizure, Complex Partial[Title/Abstract])) OR (Epileptic Seizures[Title/Abstract])) OR (Seizures, Epileptic[Title/Abstract])) OR (Epileptic Seizure[Title/Abstract])) OR (Seizure, Epileptic[Title/Abstract])) OR (Generalized Absence Seizures[Title/Abstract])) OR (Generalized Absence Seizure[Title/Abstract])) OR (Generalized Absence Seizure[Title/Abstract])) OR (Absence Seizures, Generalized[Title/Abstract])) OR (Seizure, Generalized Absence[Title/Abstract])) OR (Tonic-Clonic Seizures[Title/Abstract])) OR (Seizures, Tonic-Clonic[Title/Abstract])) OR (Generalized Tonic-Clonic Seizures[Title/Abstract])) OR (Generalized Tonic-Clonic Seizure[Title/Abstract])) OR (Generalized Tonic Clonic Seizures[Title/Abstract])) OR (Seizure, Generalized Tonic-Clonic[Title/Abstract])) OR (Seizures, Generalized Tonic-Clonic[Title/Abstract])) OR (Tonic-Clonic Seizure, Generalized[Title/Abstract])) OR (Tonic-Clonic Seizures, Generalized[Title/Abstract])) OR (Tonic Clonic Seizure[Title/Abstract])) OR (Clonic Seizures, Tonic[Title/Abstract])) OR (Clonic Seizure, Tonic[Title/Abstract])) OR (Seizure, Tonic Clonic[Title/Abstract])) OR (Tonic Clonic Seizures[Title/Abstract])) OR (Tonic-Clonic Seizure[Title/Abstract])) OR (Seizure, Tonic-Clonic[Title/Abstract])) OR (Myoclonic Seizures[Title/Abstract])) OR (Myoclonic Seizure[Title/Abstract])) OR (Seizure, Myoclonic[Title/Abstract])) OR (Clonic Seizures[Title/Abstract])) OR (Seizures, Clonic[Title/Abstract])) OR (Clonic Seizure[Title/Abstract])) OR (Seizure, Clonic[Title/Abstract])) OR (Tonic Seizures[Title/Abstract])) OR (Seizures, Tonic[Title/Abstract])) OR (Tonic Seizure[Title/Abstract])) OR (Seizure, Tonic[Title/Abstract])) OR (Atonic Seizures[Title/Abstract])) OR (Atonic Seizure[Title/Abstract])) OR (Seizure, Atonic[Title/Abstract])) OR (Convulsions[Title/Abstract])) OR (Convulsion[Title/Abstract])) OR (Absence Seizures[Title/Abstract])) OR (Petit Mal Convulsion[Title/Abstract])) OR (Convulsion, Petit Mal[Title/Abstract])) OR (Absence Seizure[Title/Abstract])) OR (Seizure, Absence[Title/Abstract]))	2,31,452	21:41:29
4	((((((((((((((((((((((((((((((((((((((((((((((((((((((((((((((((((((((((((((((((((((((((((((((((((((((((((((((((((((((((((((((("Seizures"[Mesh]) OR (Seizure[Title/Abstract])) OR (Jacksonian Seizure[Title/Abstract])) OR (Seizure, Jacksonian[Title/Abstract])) OR (Single Seizure[Title/Abstract])) OR (Seizure, Single[Title/Abstract])) OR (Single Seizures[Title/Abstract])) OR (Atonic Absence Seizures[Title/Abstract])) OR (Atonic Absence Seizure[Title/Abstract])) OR (Absence Seizure, Atonic[Title/Abstract])) OR (Absence Seizures, Atonic[Title/Abstract])) OR (Seizure, Atonic Absence[Title/Abstract])) OR (Seizures, Focal[Title/Abstract])) OR (Focal Seizure[Title/Abstract])) OR (Focal Seizures[Title/Abstract])) OR (Seizure, Focal[Title/Abstract])) OR (Partial Seizures[Title/Abstract])) OR (Partial Seizure[Title/Abstract])) OR (Seizure, Partial[Title/Abstract])) OR (Seizures, Generalized[Title/Abstract])) OR (Generalized Seizure[Title/Abstract])) OR (Generalized Seizures[Title/Abstract])) OR (Seizure, Generalized[Title/Abstract])) OR (Seizures, Sensory[Title/Abstract])) OR (Seizure, Sensory[Title/Abstract])) OR (Sensory Seizure[Title/Abstract])) OR (Sensory Seizures[Title/Abstract])) OR (Seizures, Auditory[Title/Abstract])) OR (Auditory Seizure[Title/Abstract])) OR (Auditory Seizures[Title/Abstract])) OR (Seizure, Auditory[Title/Abstract])) OR (Convulsive Seizures[Title/Abstract])) OR (Seizure, Convulsive[Title/Abstract])) OR (Seizures, Convulsive[Title/Abstract])) OR (Seizures, Motor[Title/Abstract])) OR (Motor Seizure[Title/Abstract])) OR (Motor Seizures[Title/Abstract])) OR (Seizure, Motor[Title/Abstract])) OR (Convulsive Seizure[Title/Abstract])) OR (Seizures, Gustatory[Title/Abstract])) OR (Gustatory Seizure[Title/Abstract])) OR (Gustatory Seizures[Title/Abstract])) OR (Seizure, Gustatory[Title/Abstract])) OR (Seizures, Olfactory[Title/Abstract])) OR (Olfactory Seizure[Title/Abstract])) OR (Olfactory Seizures[Title/Abstract])) OR (Seizure, Olfactory[Title/Abstract])) OR (Seizures, Somatosensory[Title/Abstract])) OR (Seizure, Somatosensory[Title/Abstract])) OR (Somatosensory Seizure[Title/Abstract])) OR (Somatosensory Seizures[Title/Abstract])) OR (Seizures, Vertiginous[Title/Abstract])) OR (Seizure, Vertiginous[Title/Abstract])) OR (Vertiginous Seizure[Title/Abstract])) OR (Vertiginous Seizures[Title/Abstract])) OR (Seizures, Vestibular[Title/Abstract])) OR (Seizure, Vestibular[Title/Abstract])) OR (Vestibular Seizure[Title/Abstract])) OR (Vestibular Seizures[Title/Abstract])) OR (Seizures, Visual[Title/Abstract])) OR (Seizure, Visual[Title/Abstract])) OR (Visual Seizure[Title/Abstract])) OR (Visual Seizures[Title/Abstract])) OR (Convulsion, Non-Epileptic[Title/Abstract])) OR (Convulsion, Non Epileptic[Title/Abstract])) OR (Convulsions, Non-Epileptic[Title/Abstract])) OR (Non-Epileptic Convulsion[Title/Abstract])) OR (Non-Epileptic Convulsions[Title/Abstract])) OR (Nonepileptic Seizures[Title/Abstract])) OR (Non-Epileptic Seizures[Title/Abstract])) OR (Non Epileptic Seizures[Title/Abstract])) OR (Nonepileptic Seizure[Title/Abstract])) OR (Seizure, Nonepileptic[Title/Abstract])) OR (Seizures, Nonepileptic[Title/Abstract])) OR (Non-Epileptic Seizure[Title/Abstract])) OR (Non Epileptic Seizure[Title/Abstract])) OR (Seizure, Non-Epileptic[Title/Abstract])) OR (Complex Partial Seizures[Title/Abstract])) OR (Complex Partial Seizure[Title/Abstract])) OR (Partial Seizure, Complex[Title/Abstract])) OR (Partial Seizures, Complex[Title/Abstract])) OR (Seizure, Complex Partial[Title/Abstract])) OR (Epileptic Seizures[Title/Abstract])) OR (Seizures, Epileptic[Title/Abstract])) OR (Epileptic Seizure[Title/Abstract])) OR (Seizure, Epileptic[Title/Abstract])) OR (Generalized Absence Seizures[Title/Abstract])) OR (Generalized Absence Seizure[Title/Abstract])) OR (Generalized Absence Seizure[Title/Abstract])) OR (Absence Seizures, Generalized[Title/Abstract])) OR (Seizure, Generalized Absence[Title/Abstract])) OR (Tonic-Clonic Seizures[Title/Abstract])) OR (Seizures, Tonic-Clonic[Title/Abstract])) OR (Generalized Tonic-Clonic Seizures[Title/Abstract])) OR (Generalized Tonic-Clonic Seizure[Title/Abstract])) OR (Generalized Tonic Clonic Seizures[Title/Abstract])) OR (Seizure, Generalized Tonic-Clonic[Title/Abstract])) OR (Seizures, Generalized Tonic-Clonic[Title/Abstract])) OR (Tonic-Clonic Seizure, Generalized[Title/Abstract])) OR (Tonic-Clonic Seizures, Generalized[Title/Abstract])) OR (Tonic Clonic Seizure[Title/Abstract])) OR (Clonic Seizures, Tonic[Title/Abstract])) OR (Clonic Seizure, Tonic[Title/Abstract])) OR (Seizure, Tonic Clonic[Title/Abstract])) OR (Tonic Clonic Seizures[Title/Abstract])) OR (Tonic-Clonic Seizure[Title/Abstract])) OR (Seizure, Tonic-Clonic[Title/Abstract])) OR (Myoclonic Seizures[Title/Abstract])) OR (Myoclonic Seizure[Title/Abstract])) OR (Seizure, Myoclonic[Title/Abstract])) OR (Clonic Seizures[Title/Abstract])) OR (Seizures, Clonic[Title/Abstract])) OR (Clonic Seizure[Title/Abstract])) OR (Seizure, Clonic[Title/Abstract])) OR (Tonic Seizures[Title/Abstract])) OR (Seizures, Tonic[Title/Abstract])) OR (Tonic Seizure[Title/Abstract])) OR (Seizure, Tonic[Title/Abstract])) OR (Atonic Seizures[Title/Abstract])) OR (Atonic Seizure[Title/Abstract])) OR (Seizure, Atonic[Title/Abstract])) OR (Convulsions[Title/Abstract])) OR (Convulsion[Title/Abstract])) OR (Absence Seizures[Title/Abstract])) OR (Petit Mal Convulsion[Title/Abstract])) OR (Convulsion, Petit Mal[Title/Abstract])) OR (Absence Seizure[Title/Abstract])) OR (Seizure, Absence[Title/Abstract])	1,48,824	21:40:19
3	"Seizures"[Mesh]	76,300	21:30:51
2	((((((((((("Epilepsy"[Mesh]) OR (Epilepsies[Title/Abstract])) OR (Seizure Disorder[Title/Abstract])) OR (Seizure Disorders[Title/Abstract])) OR (Epilepsy, Cryptogenic[Title/Abstract])) OR (Cryptogenic Epilepsies[Title/Abstract])) OR (Cryptogenic Epilepsy[Title/Abstract])) OR (Epilepsies, Cryptogenic[Title/Abstract])) OR (Aura[Title/Abstract])) OR (Auras[Title/Abstract])) OR (Awakening Epilepsy[Title/Abstract])) OR (Epilepsy, Awakening[Title/Abstract])	1,40,642	21:29:34
1	"Epilepsy"[Mesh]	1,29,415	21:28:09

Sci			
#	Search Query	Results	Date run
1	TS=(epilepsy) and Preprint Citation Index (Exclude – Database)	187480	Fri Jun 07 2024 10:43:22 GMT+0800 (中国标准时间)
2	(((((((((((((((((((((((TS=(acute epilepsy)) OR TS=(attack, epileptic)) OR TS=(cerebral seizure, epileptic)) OR TS=(chronic epilepsy)) OR TS=(comitial disease)) OR TS=(convulsion, epileptic)) OR TS=(convulsive epilepsy)) OR TS=(epilepsia)) OR TS=(epilepsy, convulsive)) OR TS=(epileptic)) OR TS=(epileptic attack)) OR TS=(epileptic convulsion)) OR TS=(epileptic disorder)) OR TS=(epileptic fit)) OR TS=(epileptic insult)) OR TS=(epileptic seizure)) OR TS=(epileptic seizure, cerebral)) OR TS=(epileptic syndrome)) OR TS=(epileptic syndromes)) OR TS=(falling sickness)) OR TS=(fit, epileptic)) OR TS=(seizure, epileptic)) OR TS=(sickness, falling)) OR TS=(tardy epilepsy) and Preprint Citation Index (Exclude – Database)	70499	Fri Jun 07 2024 10:54:10 GMT+0800 (中国标准时间)
3	#2 OR #1 and Preprint Citation Index (Exclude – Database)	199367	Fri Jun 07 2024 10:54:20 GMT+0800 (中国标准时间)
4	TS=(seizure) and Preprint Citation Index (Exclude – Database)	174688	Fri Jun 07 2024 10:54:54 GMT+0800 (中国标准时间)
5	(((TS=(seizure activities)) OR TS=(seizure activity)) OR TS=(seizure disorder)) OR TS=(seizures) and Preprint Citation Index (Exclude – Database)	174688	Fri Jun 07 2024 10:55:58 GMT+0800 (中国标准时间)
6	#4 OR #5 and Preprint Citation Index (Exclude – Database)	174688	Fri Jun 07 2024 10:56:06 GMT+0800 (中国标准时间)
7	TS=(convulsion) and Preprint Citation Index (Exclude – Database)	13864	Fri Jun 07 2024 10:57:01 GMT+0800 (中国标准时间)
8	(((((((TS=(convulsion susceptibility)) OR TS=(convulsions)) OR TS=(convulsive action)) OR TS=(convulsive disorder)) OR TS=(convulsive fit)) OR TS=(convulsive motor seizure)) OR TS=(convulsive reaction)) OR TS=(electroconvulsion) and Preprint Citation Index (Exclude – Database)	16463	Fri Jun 07 2024 10:57:59 GMT+0800 (中国标准时间)
9	#8 OR #7 and Preprint Citation Index (Exclude – Database)	16463	Fri Jun 07 2024 10:58:09 GMT+0800 (中国标准时间)
10	TS=(intestine flora) and Preprint Citation Index (Exclude – Database)	29583	Fri Jun 07 2024 10:59:06 GMT+0800 (中国标准时间)
11	((((((((((((((((((((((((((((((((((((TS=(alimentary canal flora)) OR TS=(alimentary tract flora)) OR TS=(bowel flora)) OR TS=(bowel microbiota)) OR TS=(digestive canal flora)) OR TS=(digestive tract flora)) OR TS=(enteric flora)) OR TS=(enteric microbiota)) OR TS=(flora, intestine)) OR TS=(gastro intestinal flora)) OR TS=(gastrointestinal canal flora)) OR TS=(gastrointestinal flora)) OR TS=(gastrointestinal microbiome)) OR TS=(gastrointestinal microbiota)) OR TS=(gastrointestinal tract flora)) OR TS=(gastrointestine flora)) OR TS=(gastrointestine tract flora)) OR TS=(gut bacteria)) OR TS=(gut microbiota)) OR TS=(intestinal bacteria)) OR TS=(intestinal bacterial flora)) OR TS=(intestinal bacterium)) OR TS=(intestinal canal flora)) OR TS=(intestinal flora)) OR TS=(intestinal microbe)) OR TS=(intestinal microbes)) OR TS=(intestinal microbiota)) OR TS=(intestinal microflora)) OR TS=(intestinal microorganism)) OR TS=(intestinal tract flora)) OR TS=(intestine bacteria)) OR TS=(intestine bacteria change)) OR TS=(intestine bacterial flora)) OR TS=(intestine bacterium)) OR TS=(intestine microbial flora)) OR TS=(intestine microflora)) OR TS=(intestine flora) and Preprint Citation Index (Exclude – Database)	244316	Fri Jun 07 2024 11:03:13 GMT+0800 (中国标准时间)
12	#10 OR #11 and Preprint Citation Index (Exclude – Database)	244316	Fri Jun 07 2024 11:03:20 GMT+0800 (中国标准时间)
13	#3 OR #6 OR #9 and Preprint Citation Index (Exclude – Database)	277754	Fri Jun 07 2024 11:03:37 GMT+0800 (中国标准时间)
14	#13 AND #12 and Preprint Citation Index (Exclude – Database)	537	Fri Jun 07 2024 11:03:43 GMT+0800 (中国标准时间)

### 2.2 Eligibility criteria

The inclusion criteria were as follows: (1) Control for the type of study design. (2) The study was conducted on patients with primary epilepsy. (3) The outcome metrics were gut flora abundance or gut flora richness or diversity metrics. (4) The source was published primary literature.

The exclusion criteria were as follows: (1) Animal subjects. (2) Non-epileptic subjects. (3) Secondary epilepsy such as post-traumatic epilepsy, post-stroke epilepsy, and postencephalitis epilepsy. (4) Incomplete data that prevented the extraction of raw data for statistical literature. (5) Conference papers, dissertations, and review articles. (6) Non-English papers.

### 2.3 Data extraction

The data were extracted independently by both authors from the original text or graphs. (1) General characteristics: first author, year of publication, population size, age, diagnosis. (2) Outcome metrics studied: alpha diversity (observed species index, Chao1 index, Shannon index, Simpson index, ACE index), bacterial phylum, bacterial genus.

The data were extracted directly from the original article, or the raw data were requested by email from the corresponding author of the study. In the event of a lack of response from the authors, the GetData chart digitizer was employed to procure pertinent and precise raw data that could not be obtained from the corresponding author (Liu et al., 2017).

### 2.4 Quality assessment

The potential for bias in the included studies was evaluated using a scale developed by the Agency for Healthcare Research and Quality (AHRQ) for the assessment of cross-sectional studies. Two independent authors evaluated the risk of bias based on the following criteria: (1) Is the source of data specified? (2) Whether the inclusion and exclusion criteria for the exposed and non-exposed groups were clearly defined. (3) Whether the time period for identifying patients was specified. (4) Whether the study population was consecutive. (5) Whether the assessor of subjectively-indicated patient outcomes was isolated from other objective indicators of the patient. (6) Describing any assessments made for quality assurance purposes. (7) Providing explanations for the exclusion of some patients. (8) Describing how confounders were controlled for. Each criterion could be classified as low, unclear, or high risk of bias. Two investigators conducted an independent assessment of the risk of bias and cross-validation. In the event of a discrepancy, a third party was consulted for a resolution after a discussion. The generation of random numbers using tables or computer software was deemed a low-risk method for ensuring the reliability of the random sequence. Methods deemed unreliable, such as those involving the selection of numbers artificially, were considered to pose a high risk, while those with unknown generation methods were considered to be of unclear risk. The potential for the assessor of a patient’s subjectively-derived indicators to be isolated from other objective indicators of the patient is contingent upon the rigor of the employed blinding methodology and its correct implementation. A potentially compromised blinding is defined as a failure to report all pre-specified primary outcome indicators during the study period, which is considered high risk; otherwise, it is considered low risk. The failure to report all pre-specified key outcome indicators is regarded as a high-risk factor, whereas the outcome indicators included in the fully published study plan are considered low risk.

### 2.5 Data analysis

All extracted data were converted to the same unit prior to calculation. Continuous variables were expressed as mean ± standard deviation (m = median, a = minimum, b = maximum) in accordance with the formula detailed in Supplementary Image 3 (Hozo et al., 2005). The data were analyzed using the R 4.3.3 software. The results were expressed as a Standardized Mean Difference (SMD) with a 95% confidence interval (CI). The presence of heterogeneity was evaluated through the utilization of the chi-square test and the I^2^ statistic. A *p*-value of less than 0.1 and an I^2^ value exceeding 50% were indicative of a considerable probability of heterogeneity within the data. Given the anticipated presence of heterogeneity, a random-effects model was employed. Furthermore, subgroup analyses were conducted when deemed necessary to facilitate a more comprehensive interpretation of the results. A *p*-value of less than 0.05 was considered statistically significant.

## 3 Results

### 3.1 Search results

A total of 1,254 studies were retrieved from Web of Science, PubMed, and Embase on 9 June 2024, following a comprehensive search. Two researchers employed the EndNote software to screen the titles and abstracts of the references, resulting in the removal of 363 duplicates. A total of 845 references were excluded from further consideration as they did not meet the pre-established inclusion criteria. Additionally, 46 potential references were included. Following a full-text review by two researchers, 14 studies were included in the meta-analysis (Table 2).

**Table 2:** Overall characteristics of the included literature and outcome indicators included in the literature.

References	Years	Exposed group	Age	Alpha-diversity	Phylum	Family
Türay et al. (2023)	2023	Epilepsy	7.8 ± 2.6	Shannon, simpson	–	–
Liu et al. (2023)	2023	Epilepsy	4.8 ± 2.3	Chao1	Acidobacteria Actinobacteria Bacteroidetes Cyanobacteria Firmicutes Proteobacteria Verrucomicrobia	Bifidobacterium Lachnospiracea_incertae_sedis Faecalibacterium Akkermansia Enterococcus Collinsella Streptococcus Escherichia/Shigella Veillonella Clostridium_sensu_stricto Bacteroides Klebsiella Lactobacillus Enterobacter
Cui et al. (2022)	2022	Epilepsy	30.16 ± 10.47	Chao1, Shannon, simpson, ace	Actinobacteria Bacteroidetes Proteobacteria	Escherichia/Shigella Enterobacteriaceae_unclassified Lachnospiraceae_unclassified Bifidobacterium Halomonas Bacteroides Lachnoclostridium Anaerostipes Blautia Clostridia_unclassified Dorea Roseburia Megamonas Erysipelotrichaceae_UCG-003
Dong et al. (2022)	2022	Epilepsy	33.1 ± 9.24	Chao1	–	–
Dai et al. (2022)	2022	Drug-resistant epilepsy, Non-drug-resistant epilepsy	25.46 ± 11.36	Shannon	–	–
Zhou et al. (2022)	2022	Focal epilepsy	6.38 ± 2.01	Chao1, Shannon, simpson, ace	Actinobacteria	Escherichia/Shigella Faecalibacterium
Xu et al. (2021)	2021	WEST syndrome	–	Chao1, Shannon	Firmicutes Actinobacteria Proteobacteria Bacteroidetes Verrucomicrobia	Bifidobacterium Bacteroides Escherichia/Shigella Veillonella Streptococcus Lachnospiraceae Klebsiella Clostridium_sensu_stricto Enterococcus Akkermansia Collinsella Faecalibacterium Lactobacillus
Gong et al. (2021)	2021	Refractory epilepsy in children	27.41 ± 8.17	Chao1, simpson	–	Akkermansia Bifidobacterium Enterococcus Actinomyces Subdoligranulum Dialister Alloprevotella Ruminococcaceae
Wan et al. (2021)	2021	WEST syndrome	8.35 ± 3.36	Shanon, simpson	–	Bifidobacterium Veillonella Bacteroides Streptococcus Prevotella Enterococcus Lactobacillus Megasphaera Roseburia
Gong et al. (2020)	2020	Epilepsy	27.42 ± 8.17	Chao1, Shannon, simpson	Firmicutes Actinobacteria Verrucomicrobia Cyanobacteria Nitrospirae	Blautia Bifidobacterium Subdoligranulum Parabacterioides Akkermansia Dialister Eubacterium Dorea Anaerostipes
Lee et al. (2020)	2020	Epilepsy	3.37 ± 1.50	Chao1, Shannon, simpson, ace	–	–
Lindefeldt et al. (2019)	2019	Refractory epilepsy in children	–	Chao1, shannon	Actinobacteria Bacteroidetes Firmicutes Proteobacteria	Bifidobacterium etc.
Peng et al. (2018)	2018	Drug-resistant epilepsy	28.9 ± 11.75	Chao1	–	–
		Non-drug-resistant epilepsy	27.25 ± 12.92	Chao1	–	–
Xie et al. (2017)	2017	Epilepsy	–	Shannon	–	–

–, Not mentioned in relevant literature or data not available.

### 3.2 Characteristics of the selected studies

The experimental group was diagnosed with epilepsy in 14 studies, eight of which involved children. In five studies, the mean age of the experimental group was less than 18 years, while in seven studies, it was greater than 18 years. In two studies, the mean age could not be calculated due to the lack of specification regarding the age of the control group.

Six studies did not specify the type of epilepsy. Two studies focused on refractory epilepsy in children, two studies investigated drug-resistant and drug-sensitive epilepsy, two studies explored West’s syndrome, one study examined focal epilepsy, and the cause of epilepsy in one study was unclear.

The six principal outcome indicators are alpha diversity (Chao1 index, Shannon’s index, Simpson’s index, and Ace’s index), phylum, and genus. These are presented in Table 2.

### 3.3 Risk of bias assessment

All studies were deemed to have a low risk of bias in terms of the source of information, inclusion and exclusion criteria, assessment of quality valuation, explanation for exclusion of each individual patient, and measures to control for confounders. Thirteen studies were identified as being at high risk of bias with regard to the question of whether the assessor of the patient’s subjectivized indicator was isolated from the patient’s other objective indicators. In addition, some of the studies were at high risk of bias with respect to whether the subjects were consecutive or not, and whether a time period for identifying the patients was provided. The risk of bias was unclear in terms of whether the subjects were consecutive or not, quality assessment, and control of confounders. In some cases, the risk of bias was unclear.

**Figure 2:**
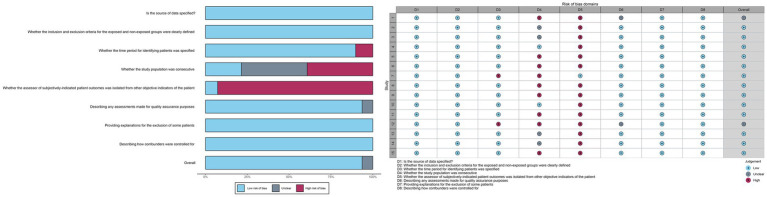
Risk of bias assessment chart.

The overall assessment of risk of bias was low in 11 trials and uncertain in 2 trials (Figure 2).

### 3.4 Differential assessment of flora outcomes in epileptic and non-epileptic patients

#### 3.4.1 Alpha diversity

**Figure 3:**
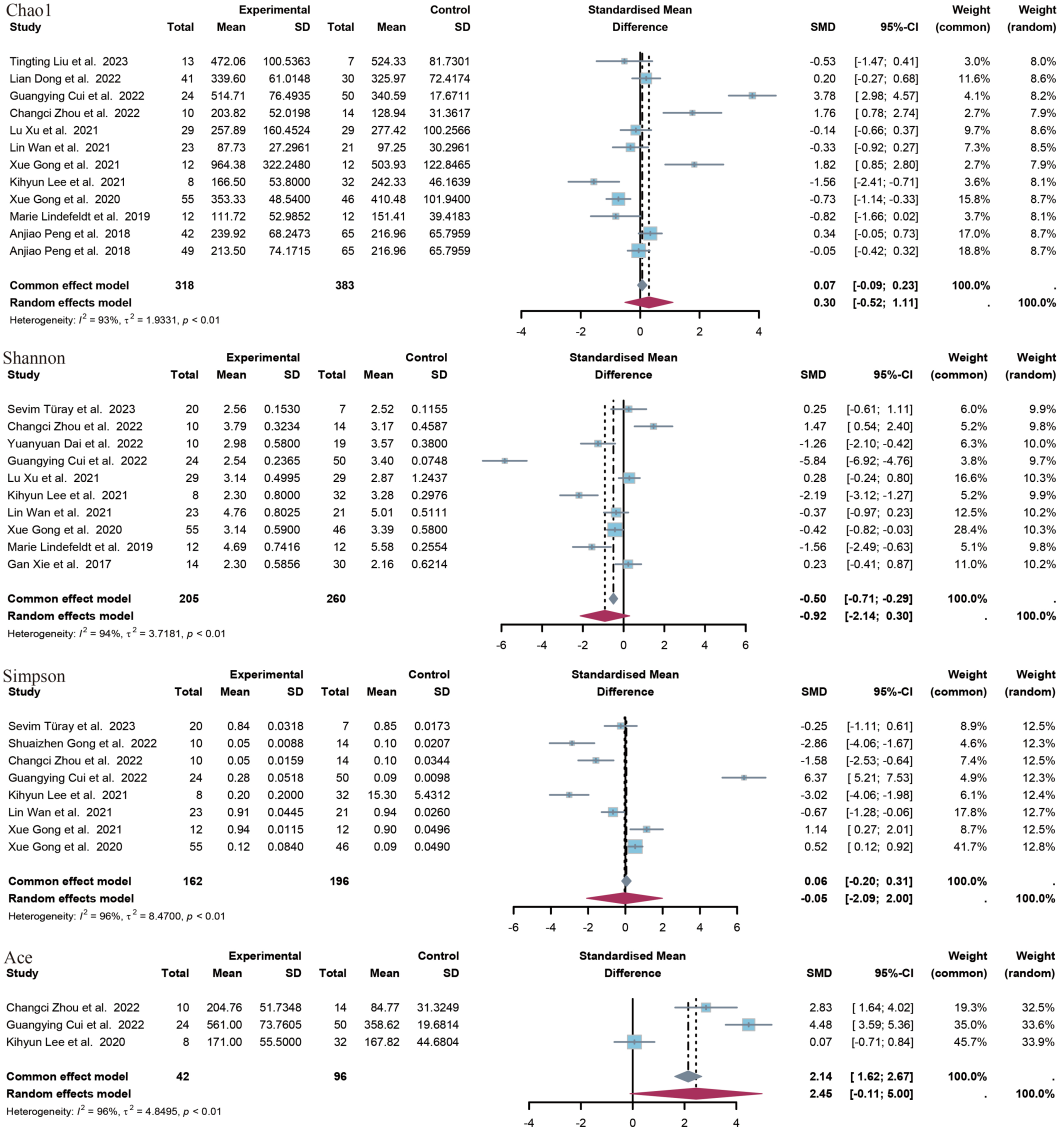
Forest plot of alpha diversity index.

In this study, a range of alpha diversity indices were employed to evaluate microbial diversity within a given group. These included estimates of richness (such as the observed species index and the Chao1 index) and indices that combine richness and evenness (such as the Shannon index, the Simpson index and the ACE index) (Figure 3).

A total of 12 controlled trials provided data on the Chao1 index of richness. Of these, five trials revealed a significant difference between the epilepsy group and the control group with regard to the Chao1 index. The meta-analysis of this study revealed no significant difference in the SMD of the species index Chao1 (SMD = 0.30, *p* = 0.47, 95% CI = −0.52 to 1.11, I^2^ = 93%). A subgroup analysis according to age revealed no significant difference between minors (aged <18 years) and adults. The former exhibited a standardized mean difference (SMD) of −0.18 (*p* = 0.73, 95% CI = −1.18 to 0.83, I^2^ = 85%), while the latter displayed a SMD of 0.63 (*p* = 0.30, 95% CI = −56 to 1.86, I^2^ = 95) (Supplementary Images 1, 2).

A total of 10 studies provided data on Shannon’s index, and in seven of these studies, a significant difference was observed between the epilepsy group and the control group. In all five studies showing a significant reduction in Shannon’s index in the epilepsy group, compared to the control group, the opposite was true in two studies. The results of the meta-analysis demonstrated no statistically significant difference in the SMD of the Shannon index (SMD = −0.92, *p* = 0.14, 95% CI = −2.14 to 0.30, I^2^ = 94%), nor in the subgroup analyses (minors: SMD = −0.11, *p* = 0.85, 95% CI = −1.24 to 1.03, I^2^ = 88%). The SMD was −1.74 (*p* = 0.10), with a 95% CI of −3.80 to 0.32 and an I^2^ value of 96%.

Seven studies provided data on Simpson’s index. Five studies demonstrated a statistically significant difference between patients with epilepsy and a control group. Three of these studies showed a significant increase in Simpson’s index in the epilepsy group compared to the control group, while the other two studies yielded opposite results. The meta-analysis of the present study revealed no statistically significant difference in Simpson’s index between patients with epilepsy and the healthy population [standardized mean difference (SMD) 0.05, *p* = 0.96, 95% confidence interval (CI) −2.09 to 2.00, I^2^ = 96.5%]. The ACE index was provided as an indicator of outcome by only three studies. The meta-analysis of these studies showed a non-significant difference (SMD = 2.45, *p* = 0.06, 95% CI = −0.11 to 5.00, I^2^ = 96.4%).

#### 3.4.2 Phylum of bacteria

**Figure 4:**
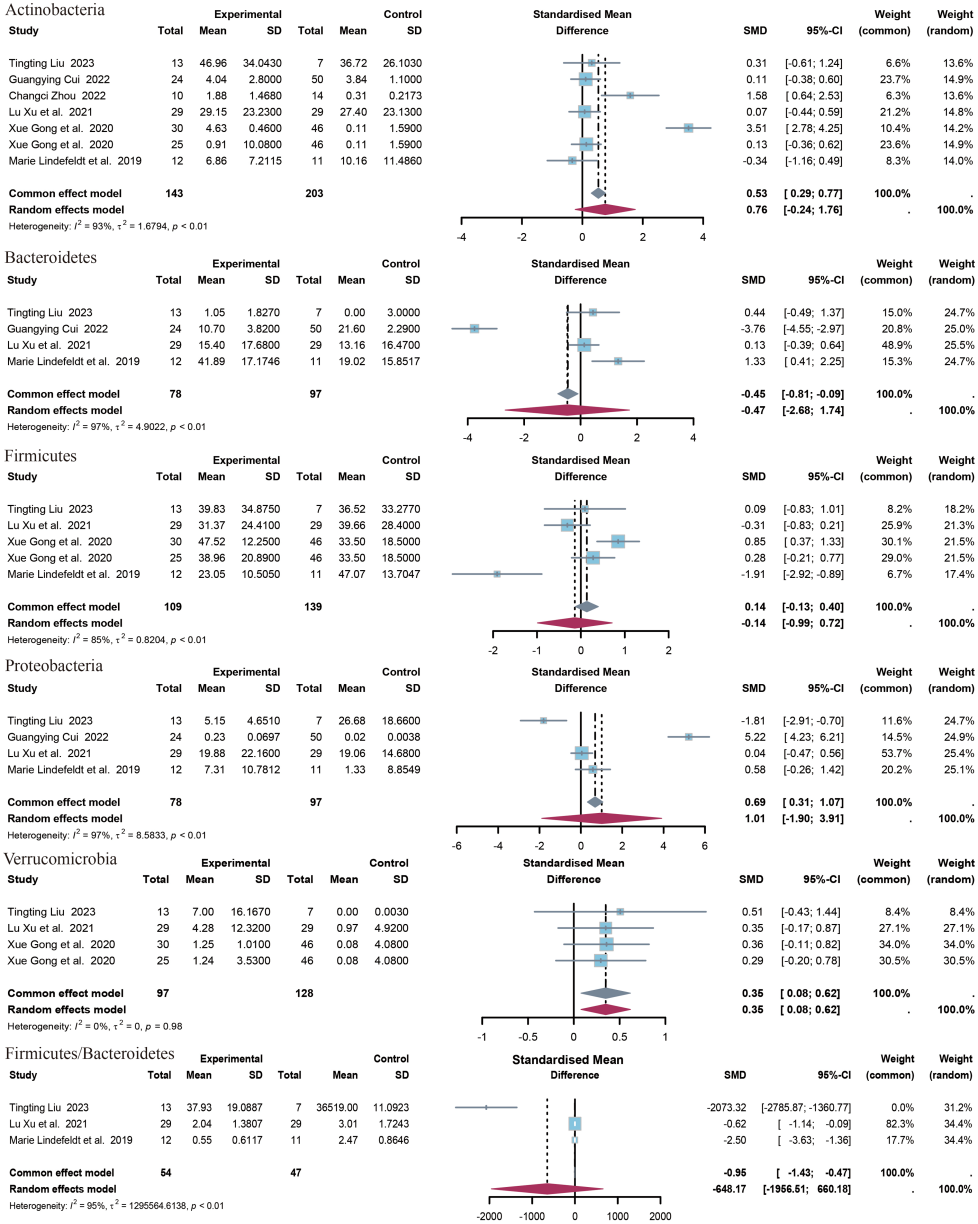
Forest plot of the phylum level.

The present study identified five distinct clades (Figure 4). The identified clades were *Bacteroidetes*, *Actinobacteria*, *Verrucomicrobia*, *Firmicutes*, and *Proteobacteria*. Additionally, the *Firmicutes/Bacteroidetes* (F/B) ratio was included in the outcome index. The results of the meta-analysis indicated that there was a statistically significant association between *Verrucomicrobia* and the outcome variable (SMD = 0.35, 95% CI 0.08 to 0.62, *p* = 0.01, I^2^ = 0%). No statistically significant differences were observed in the levels or F/B ratios for the remaining clades. Only data on *Acidobacteria* levels were available from the study by Liu et al. (2023), which demonstrated a significant difference between the upper epilepsy group and the control population (SMD = 0.35, *p* = 0.003, 95% CI = −2.68 to −0.54).

#### 3.4.3 Genera of bacteria

**Figure 5:**
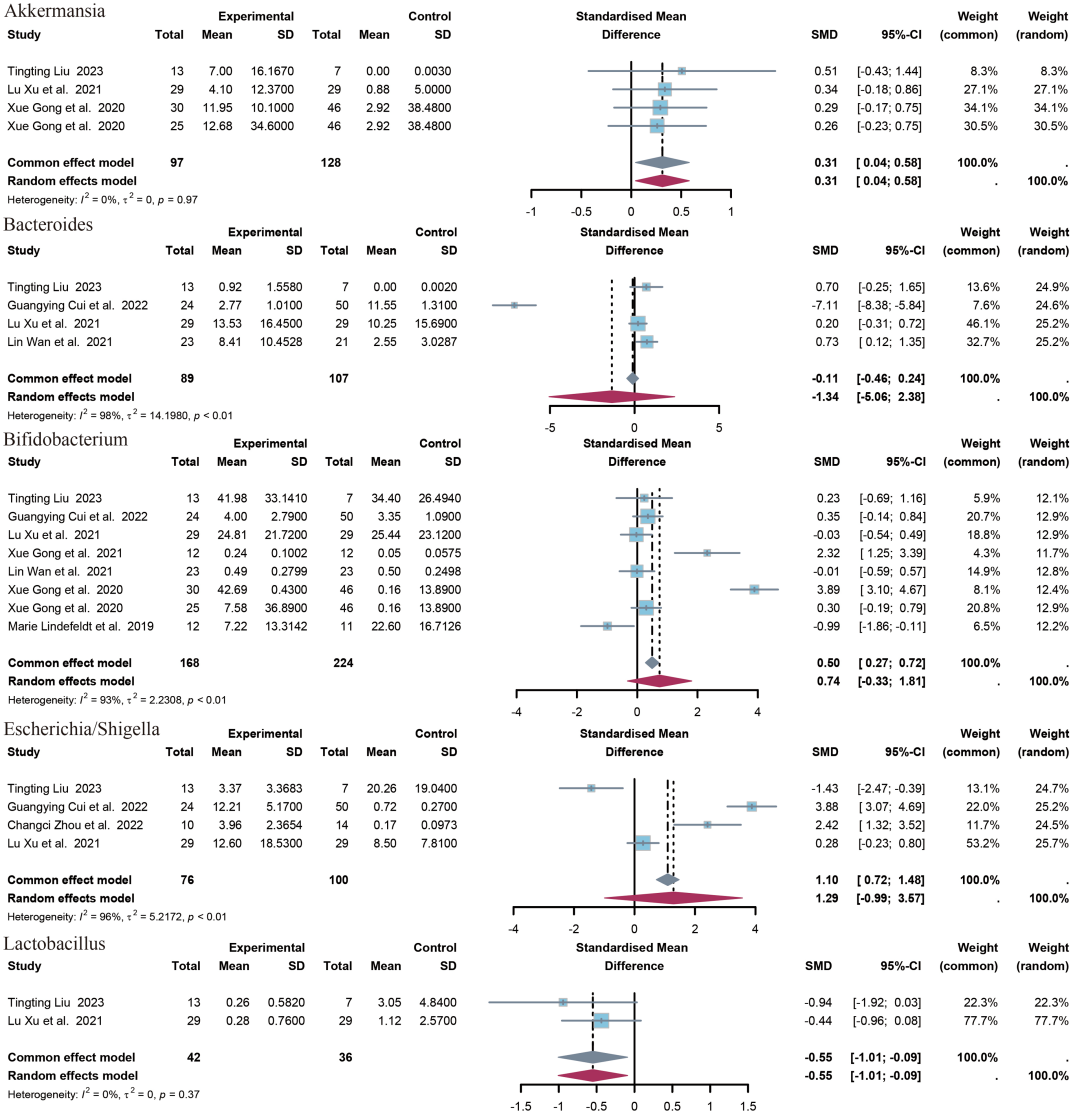
Forest plot of the genus level.

A total of 14 genera have been identified. The identified genera are as follows: *Bifidobacterium*, *Faecalibacterium*, *Akkermansia*, *Enterococcus*, *Collinsella*, *Streptococcus*, *Escherichia/Shigella*, *Veillonella*, *Clostridium sensu strict*, *Bacteroides*, *Klebsiella, Lactobacillus*, *Enterobacter*. Significant differences were observed in the abundance of *Akkermansia* (SMD = 0.31, *p* = 0.02, 95% CI = −0.04 to 0.58, I^2^ = 0%) and *Lactobacillus* (SMD = −0.55, *p* = 0.02, 95% CI = −1.10 to 0.09, I^2^ = 0%) between epileptic patients and controls (Figure 5).

**Figure 6:**
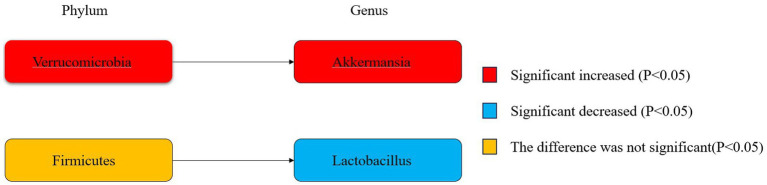
Phylogenetic characterisation.

Figure 6 illustrates the phylogenetic characteristics of the abundance of taxonomic differences between epileptic and normal populations at the phylum, order and genus levels, with colors indicating different bacterial variants. The colors red, blue and orange indicate increased abundance, decreased abundance and inconsistent variation, respectively.

## 4 Discussion

In order to identify changes in the diversity, abundance, and homogeneity of the gut microbiota after a diagnosis of epilepsy, as well as possible characteristics of bacteria that reduce seizures, three of the most commonly used databases were searched.

### 4.1 About alpha-diversity

**Figure 7:**
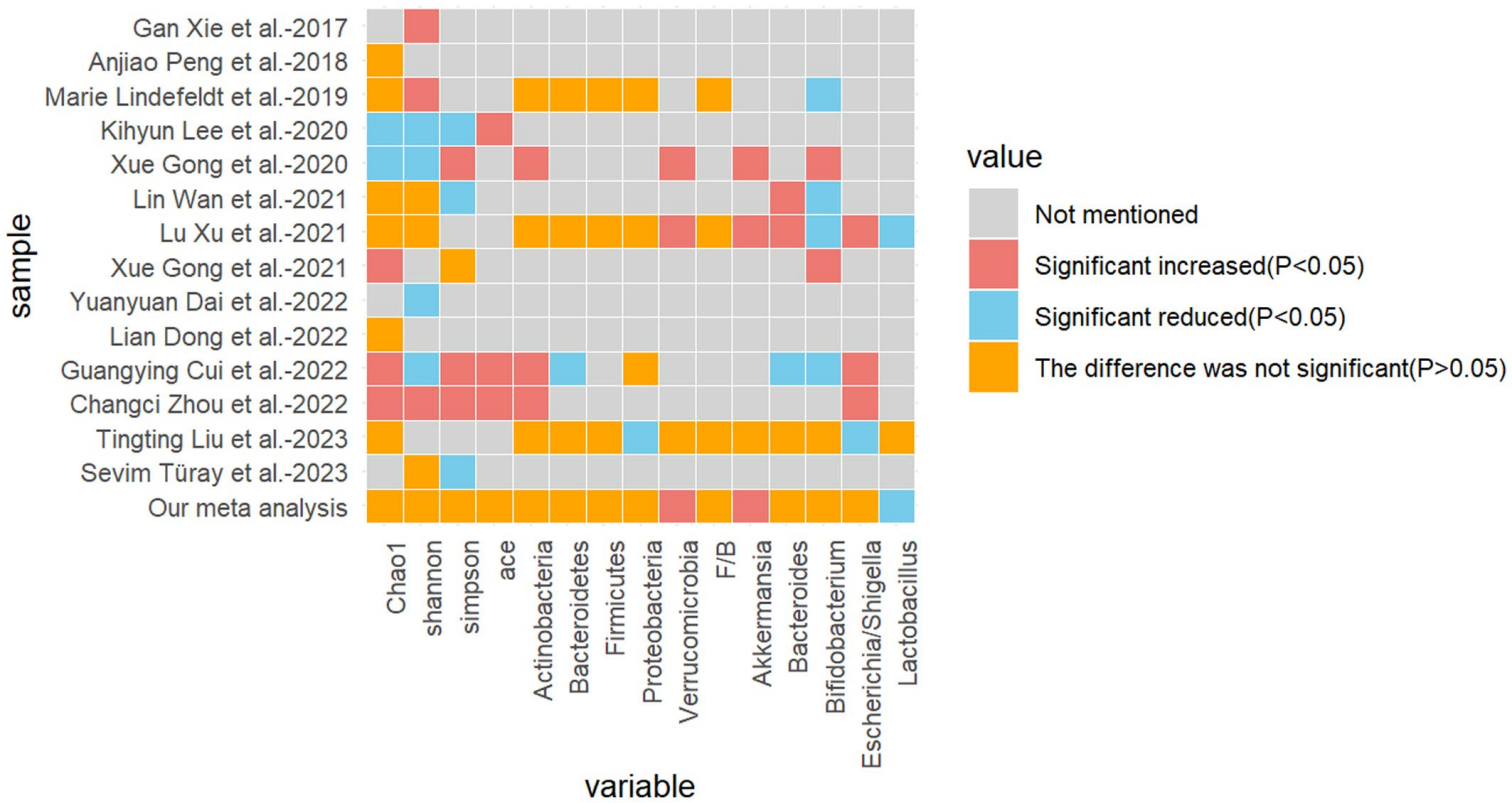
Heatmap of the difference between the included studies and the relevant metrics.

The present study encompasses 14 high-quality studies (Figure 7), encompassing alpha diversity and relative abundance at the phylum and genus levels. Alpha diversity is associated with the number of species in a local, homogeneous habitat, also referred to as intra-habitat diversity. In the context of the human gut flora, higher alpha diversity is typically indicative of a more complex and diverse microbial community, exhibiting greater metabolic versatility and stability, and a healthier metabolic profile, particularly with regard to drug metabolism (Li et al., 2022; Knight et al., 2018). It is also a marker of intestinal health. The Chao1 index, the Shannon index, the Simpson index, and the ACE index are frequently employed as measures of alpha diversity. The Chao1 index is of particular significance in that it is capable of deriving a theoretical abundance from the number of species observed, which is closer to the true abundance. Furthermore, it is particularly sensitive to rare species. The Shannon index is of significance in that it quantifies the uncertainty in the diversity of the community, that is to say, the difficulty of predicting the species identity of an individual selected at random. The Simpson index is of significance in that it reflects the degree of dominance and the even distribution of species diversity in the community (Supplementary Presentation 1). In a previous study of gut flora in epileptic patients, Zhou et al. (2022) demonstrated that the alpha diversity of the epileptic group differed significantly from that of the healthy control group, with a notable decrease in the Shannon index and an increase in the Simpson index. Similarly, Xie et al. (2017) reported that the alpha diversity of gut flora in infants with refractory epilepsy was reduced in comparison to healthy subjects. Additionally, Xu et al. (2021) and Gong et al. (2021) demonstrated that there were no statistically significant differences between the refractory epilepsy group and the refractory epilepsy group after ketogenic diet treatment compared to healthy controls. However, the study did indicate a potential trend toward a decrease. Another study by the same author revealed that the alpha diversity index of children in the epilepsy group was significantly lower than that of the family control group (Gong et al., 2020). The results were inconclusive due to the heterogeneity of the samples, the diversity of the experimental approaches, the variability in the etiology of epilepsy, the differing sensitivities to antiepileptic drugs, and the age range of the participants. It is therefore recommended that further studies be conducted using more specialized experiments with larger sample sizes.

### 4.2 About levels of genus and phylum

This study examines characteristic gut microbiota taxa by selecting flora from multiple studies with sufficient data for meta-analysis. The findings of different studies have yielded disparate conclusions, with some studies even reaching completely opposite conclusions. The two most abundant phyla are *Anabaena* and *Firmicutes* (Zhang et al., 2023). A significant decrease in Firmicutes was observed in a study by Cui et al. (2022). This group is complex, comprising intricate sub-phylum, orders, and genera under the genus *Firmicutes*. Additionally, relatively few studies have been conducted on this group. The *Firmicutes/Bacteroidetes* (F/B) ratio is regarded as a key indicator for depicting intestinal disorders (Mohamed Qadir and Assafi, 2021). Prior research has revealed an elevated F/B ratio in the intestinal microbiota of individuals diagnosed with epilepsy (Arulsamy et al., 2020), indicating a potential correlation between this alteration in microbiota structure and the production of short-chain fatty acids, which may contribute to the development of severe inflammation and complex infections (Bazzocchi et al., 2021; Rodenhouse et al., 2022). However, in the meta-analysis of the present study, the F/B ratio was not significantly different in the epileptic and control groups, which may be related to the small sample size. In this study, there was no significant difference between epileptic patients and healthy controls in the phylum Bacteroidetes, but *Lactobacillus* under *Bacteroidetes* was significantly reduced in the group of epileptic patients, and *Lactobacillus* is able to regulate the balance of the intestinal microbial community, promote the digestion of food and absorption of nutrients, reduce the cholesterol level of the body, and alleviate the intestinal inflammation and infection,. It is also implied that there exists the possibility of decreased intestinal anti-inflammatory capacity in epilepsy patients (Schloissnig et al., 2013). The results of the present study showed a significant increase in *Verrucomicrobia* and subordinate *Akkermansia* in the intestinal microbiota of epileptic patients compared to healthy controls. The abundance of *Verrucomicrobia* is closely related to intestinal health, contributing to glucose homeostasis in the human gut; and also possesses anti-inflammatory properties, which may further aid in intestinal health, as demonstrated by studies showing that a positive correlation between *Verrucomicrobia* and the foxp3 gene, a gene that expresses anti-inflammatory and immunity properties in humans (Yan et al., 2023). *Akkermansia*, which is under the phylum of *Verrucomicrobia*, is present in large quantities under normal conditions and is considered to be used as a probiotic (Zhai et al., 2019). The relative abundance of *Akkermansia* is reduced in glucose metabolism, obesity and inflammatory bowel disease (Cani and de Vos, 2017; Rajilić-Stojanović et al., 2013), and studies have shown (Zhai et al., 2019; Derrien et al., 2017) that it promotes intestinal barrier integrity, increases mucus thickness, and is conducive to metabolic and immune responses. The precise mechanism of interaction between *Akkermansia* and epilepsy remains unclear. Olson et al. (2018) showed that in a mouse model of refractory epilepsy, *Akkermansia* mediated a ketogenic diet-induced protective effect against 6-hz-induced seizures. However, the results of a Mendelian randomized study on epilepsy risk and intestinal flora (Zeng et al., 2023) showed that *Akkermansia* was able to increase the risk of epilepsy. In conclusion, *Akkermansia* has both a protective effect and a pathogenic risk, and therefore its study needs to be further deepened to clarify its specific mechanism of action.

### 4.3 Interaction of the gut-brain axis

The experimental results included in this study pertain to the changes in gut microbiology that occur subsequent to the diagnosis of epilepsy. However, the specific causal relationship between seizure and gut flora remains unclear. It has been proposed that the gut microbiome may play a role in the etiology of epilepsy (Gong et al., 2020). Medel-Matus et al. demonstrated that altered gut microbiome can trigger seizures by transplanting feces from epileptic mice into non-epileptic mice (Medel-Matus et al., 2018). Lum et al. explored the potential mechanisms of the gut microbiome in the treatment of epilepsy by investigating the effects of a ketogenic diet on the gut microbiome in epileptic patients (Lum et al., 2020). Animal and human studies have demonstrated that *γ*-aminobutyric acid (GABA) plays a pivotal role in the alteration of intestinal flora in relation to seizures in temporal lobe epilepsy (Goldberg and Coulter, 2013; Avoli et al., 2016). Additionally, these studies have indicated that the intestinal flora may alter neurotransmitter content to facilitate brain-gut interactions via the vagus nerve. Lactobacillus has been demonstrated to modulate mood and central GABA receptor levels via sympathetic nerves (Bravo et al., 2011). It is possible that intestinal flora and their products modulate sympathetic activity by influencing the secretion of enteroendocrine cells (Bonaz et al., 2018). Furthermore, epigenetic modifications of genes have been identified as a contributing factor in the pathogenesis and anti-epileptic mechanisms of epilepsy (Younus and Reddy, 2017). This process is regulated by metabolites associated with gut flora (Reddy et al., 2018). Abnormalities in immune regulation represent a significant contributing factor to seizure. Additionally, immune disorders have the potential to induce alterations in the gut commensal flora (Weis and Round, 2021), suggesting a potential convergence between seizure and gut disorders as a consequence of abnormalities in immune regulation.

The current research on gut flora is primarily concerned with the pathogenesis of various conditions, with treatments for several diagnoses of epilepsy including ketogenic diets, fecal transplants, antibiotic therapy, probiotic supplementation, prebiotics, and interventions targeting antiepileptic drug-flora interactions (Liu et al., 2023; Dahlin and Prast-Nielsen, 2019; Ułamek-Kozioł et al., 2019; Mejía-Granados et al., 2021; Amlerova et al., 2021). Over the past decade, research in the field of gut flora has flourished, resulting in significant advancements. However, it is crucial to acknowledge the intricate composition of the intestinal flora and the intestinal immune environment, as well as their distinctive characteristics across different individuals. This indicates the necessity for caution when extrapolating findings from experimental animals to humans. Firstly, although animal models (e.g., mice) are a valuable resource in research, they differ from humans in terms of immune response, gut structure, etc., which may affect the translation from animal models to humans (Schloissnig et al., 2013; Kaiko et al., 2016; Falony et al., 2016). Secondly, the human gut microbiome comprises over 1,000 microorganisms, with intricate interactions between these microorganisms and their host (Mabwi et al., 2020). This complexity gives rise to challenges in the precise regulation and application of the microbiome. Moreover, it is essential to consider the inter-individual variability observed across different ethnic, gender, and age groups. It is therefore evident that a significant future development in the exploration and improvement of epilepsy treatments will be the investigation of the changes in gut flora that can have a considerable impact on complex populations with significant heterogeneity. This will facilitate a more precise understanding of the interactions between gut flora and health and disease, thereby providing a scientific basis for the development of personalized therapeutic regimens. The last, when translating findings from animal models to human therapeutic applications, it is crucial to ensure the safety and efficacy of the treatment. This necessitates the undertaking of extensive clinical studies and validation.

## 5 Limitation

It should be noted that this meta-analysis is not without limitations. Firstly, most studies include experimental analyses regarding beta diversity. Since principal component analysis (PCA) is currently the main method of beta analysis, which selects the eigenvectors corresponding to the top few largest eigenvalues based on the magnitude of the eigenvalues, and these eigenvectors are known as the principal components, however, the eigenvalues of the top few eigenvalues of each study were mixed, so meta-analyses were not performed in this study at the level of beta analysis. Secondly, the study included different populations, including geographic location, gender, weight and age. As a result, it was not possible to accurately assess the effect of these confounding factors on the outcome indicators. Thirdly, the length of time the sample was collected was not known. As a consequence, it was not possible to assess the effect of this factor on the outcome indicators. The initial intention of this study was to subgroup all indicators according to age in order to more effectively illustrate the results. However, due to the heterogeneity of the available data, only subgroup analyses were performed for some of the outcomes.

## 6 Conclusion

The meta-analysis of this study demonstrated that the alteration in alpha-diversity within the intestinal flora of patients with epilepsy was not statistically significant when compared to controls. However, there was a notable increase in *Verrucomicrobia* and its subordinate *Akkermansia*. Furthermore, a notable reduction in Lactobacillus was observed. It is postulated that epilepsy exerts a relatively minor impact on the abundance, homogeneity, and diversity of the intestinal microbiota in patients, whereas a more pronounced influence is exerted on the alteration of the relative abundance of the flora. Furthermore, the collective findings of previous studies indicate that *Lactobacillus* may confer beneficial effects. In contrast, *Akkermansia* are understood to play a complex and multifaceted role, with characteristics that may be two-faced. Given the considerable heterogeneity and restricted sample size, it is imperative to conduct more rigorous methodological studies.

## Data Availability

The original contributions presented in the study are included in the article/Supplementary material, further inquiries can be directed to the corresponding author.

## References

[ref1] Aaberg K. M. Gunnes N. Bakken I. J. Lund Søraas C. Berntsen A. Magnus P. . (2017). Incidence and prevalence of childhood epilepsy: a Nationwide cohort study. Pediatrics 139:e20163908. doi: 10.1542/peds.2016-3908 28557750

[ref2] Amlerova J. Šroubek J. Angelucci F. Hort J. (2021). Evidences for a role of gut microbiota in pathogenesis and management of epilepsy. Int. J. Mol. Sci. 22:5576. doi: 10.3390/ijms22115576, PMID: 34070389 PMC8197531

[ref3] Arulsamy A. Tan Q. Y. Balasubramaniam V. O'Brien T. J. Shaikh M. F. (2020). Gut microbiota and epilepsy: a systematic review on their relationship and possible therapeutics. ACS Chem. Neurosci. 11, 3488–3498. doi: 10.1021/acschemneuro.0c00431, PMID: 33064448

[ref4] Avoli M. de Curtis M. Gnatkovsky V. Gotman J. Köhling R. Lévesque M. . (2016). Specific imbalance of excitatory/inhibitory signaling establishes seizure onset pattern in temporal lobe epilepsy. J. Neurophysiol. 115, 3229–3237. doi: 10.1152/jn.01128.2015, PMID: 27075542 PMC4946603

[ref5] Bazzocchi G. Turroni S. Bulzamini M. C. D’Amico F. Bava A. Castiglioni M. . (2021). Changes in gut microbiota in the acute phase after spinal cord injury correlate with severity of the lesion. Sci. Rep. 11:12743. Published 2021 Jun 17. doi: 10.1038/s41598-021-92027-z, PMID: 34140572 PMC8211659

[ref6] Bonaz B. Bazin T. Pellissier S. (2018). The Vagus nerve at the Interface of the microbiota-gut-brain Axis. Front. Neurosci. 12:49. doi: 10.3389/fnins.2018.00049, PMID: 29467611 PMC5808284

[ref7] Bravo J. A. Forsythe P. Chew M. V. Escaravage E. Savignac H. M. Dinan T. G. . (2011). Ingestion of Lactobacillus strain regulates emotional behavior and central GABA receptor expression in a mouse via the vagus nerve. Proc. Natl. Acad. Sci. USA 108, 16050–16055. doi: 10.1073/pnas.1102999108, PMID: 21876150 PMC3179073

[ref8] Cani P. D. de Vos W. M. (2017). Next-generation beneficial microbes: the case of *Akkermansia muciniphila*. Front. Microbiol. 8:1765. doi: 10.3389/fmicb.2017.01765, PMID: 29018410 PMC5614963

[ref9] Cui G. Liu S. Liu Z. Chen Y. Wu T. Lou J. . (2022). Gut microbiome distinguishes patients with epilepsy from healthy individuals. Front. Microbiol. 12:696632. doi: 10.3389/fmicb.2021.696632, PMID: 35069460 PMC8777111

[ref10] Dahlin M. Prast-Nielsen S. (2019). The gut microbiome and epilepsy. EBioMedicine 44, 741–746. doi: 10.1016/j.ebiom.2019.05.024, PMID: 31160269 PMC6604367

[ref11] Dai Y. Wang M. Zhong D. Xu X. (2022). *Bacillus subtilis* plays a role in the inhibition of transporter ABCB1 in Caco-2 cells. Epilepsy Res. 183:106925. doi: 10.1016/j.eplepsyres.2022.106925, PMID: 35526327

[ref12] Derrien M. Belzer C. de Vos W. M. (2017). Akkermansia muciniphila and its role in regulating host functions. Microb. Pathog. 106, 171–181. doi: 10.1016/j.micpath.2016.02.005 26875998

[ref13] Dong L. Zheng Q. Cheng Y. Zhou M. Wang M. Xu J. . (2022). Gut microbial characteristics of adult patients with epilepsy. Front. Neurosci. 16:803538. doi: 10.3389/fnins.2022.803538, PMID: 35250450 PMC8888681

[ref14] Elliott J. DeJean D. Clifford T. Coyle D. Potter B. K. Skidmore B. . (2019). Cannabis-based products for pediatric epilepsy: a systematic review. Epilepsia 60, 6–19. doi: 10.1111/epi.14608, PMID: 30515765

[ref15] Falony G. Joossens M. Vieira-Silva S. Wang J. Darzi Y. Faust K. . (2016). Population-level analysis of gut microbiome variation. Science 352, 560–564. doi: 10.1126/science.aad3503, PMID: 27126039

[ref16] Fisher R. S. Acevedo C. Arzimanoglou A. Bogacz A. Cross J. H. Elger C. E. . (2014). ILAE official report: a practical clinical definition of epilepsy. Epilepsia 55, 475–482. doi: 10.1111/epi.12550, PMID: 24730690

[ref17] Fisher R. S. Cross J. H. D'Souza C. French J. A. Haut S. R. Higurashi N. . (2017). Instruction manual for the ILAE 2017 operational classification of seizure types. Epilepsia 58, 531–542. doi: 10.1111/epi.13671 28276064

[ref18] Fisher R. S. Cross J. H. French J. A. Higurashi N. Hirsch E. Jansen F. E. . (2017). Operational classification of seizure types by the international league against epilepsy: position paper of the ILAE Commission for Classification and Terminology. Epilepsia 58, 522–530. doi: 10.1111/epi.13670, PMID: 28276060

[ref19] Gacesa R. Kurilshikov A. Vich Vila A. Sinha T. Klaassen M. A. Y. Bolte L. A. . (2022). Environmental factors shaping the gut microbiome in a Dutch population. Nature 604, 732–739. doi: 10.1038/s41586-022-04567-7 35418674

[ref20] Goldberg E. M. Coulter D. A. (2013). Mechanisms of epileptogenesis: a convergence on neural circuit dysfunction. Nat. Rev. Neurosci. 14, 337–349. doi: 10.1038/nrn3482, PMID: 23595016 PMC3982383

[ref21] Gong X. Cai Q. Liu X. An D. Zhou D. Luo R. . (2021). Gut flora and metabolism are altered in epilepsy and partially restored after ketogenic diets. Microb. Pathog. 155:105832. doi: 10.1016/j.micpath.2022.105832 33894293

[ref22] Gong X. Liu X. Chen C. Lin J. Li A. Guo K. . (2020). Alteration of gut microbiota in patients with epilepsy and the potential index as a biomarker. Front. Microbiol. 11:517797. doi: 10.3389/fmicb.2020.517797, PMID: 33042045 PMC7530173

[ref23] Hozo S. P. Djulbegovic B. Hozo I. (2005). Estimating the mean and variance from the median, range, and the size of a sample. BMC Med. Res. Methodol. 5:13. Published 2005. doi: 10.1186/1471-2288-5-13, PMID: 15840177 PMC1097734

[ref24] Kaiko G. E. Ryu S. H. Koues O. I. Pearce E. L. Oltz E. M. Stappenbeck T. S. . (2016). The colonic crypt protects stem cells from microbiota-derived metabolites [published correction appears in cell. 2016; 167(4): 1137. Doi:10.1016/j.cell.2016.10.034]. Cell 165, 1708–1720. doi: 10.1016/j.cell.2016.05.018 27264604 PMC5026192

[ref25] Knight R. Vrbanac A. Taylor B. C. Aksenov A. Callewaert C. Debelius J. . (2018). Best practices for analysing microbiomes. Nat. Rev. Microbiol. 16, 410–422. doi: 10.1038/s41579-018-0029-9 29795328

[ref26] Kwan P. Brodie M. J. (2000). Early identification of refractory epilepsy. N. Engl. J. Med. 342, 314–319. doi: 10.1056/NEJM200002033420503 10660394

[ref27] Lee K. Kim N. Shim J. O. Kim G. H. (2020). Gut bacterial dysbiosis in children with intractable epilepsy. J. Clin. Med. 10:5. doi: 10.3390/jcm10010005 33375063 PMC7792797

[ref28] Li Z. Zhou J. Liang H. Ye L. Lan L. Lu F. . (2022). Differences in alpha diversity of gut microbiota in neurological diseases. Front. Neurosci. 16:879318. doi: 10.3389/fnins.2022.879318 35837118 PMC9274120

[ref29] Lindefeldt M. Eng A. Darban H. Bjerkner A. Zetterström C. K. Allander T. . (2019). The ketogenic diet influences taxonomic and functional composition of the gut microbiota in children with severe epilepsy. NPJ Biof. Microb. 5:5. doi: 10.1038/s41522-018-0073-2, PMID: 30701077 PMC6344533

[ref30] Liu T. Jia F. Guo Y. Wang Q. Zhang X. Chang F. . (2023). Altered intestinal microbiota composition with epilepsy and concomitant diarrhea and potential indicator biomarkers in infants. Front. Microbiol. 13:1081591. doi: 10.3389/fmicb.2022.1081591 36713168 PMC9874329

[ref31] Liu H. Wu H. Yao C. . (2017). Advanced methods of data extraction for continuous outcomes in meta-analysis. Chin. J. Evid. Based Med. 17, 117–121. doi: 10.7507/1672-2531.201612004

[ref32] Lum G. R. Olson C. A. Hsiao E. Y. (2020). Emerging roles for the intestinal microbiome in epilepsy. Neurobiol. Dis. 135:104576. doi: 10.1016/j.nbd.2019.104576 31445165

[ref33] Mabwi H. A. Kim E. Song D. G. Yoon H. S. Pan C. H. Komba E. V. G. . (2020). Synthetic gut microbiome: advances and challenges. Comput. Struct. Biotechnol. J. 19, 363–371. doi: 10.1016/j.csbj.2020.12.029, PMID: 33489006 PMC7787941

[ref34] Medel-Matus J. S. Shin D. Dorfman E. Sankar R. Mazarati A. (2018). Facilitation of kindling epileptogenesis by chronic stress may be mediated by intestinal microbiome. Epilepsia Open. 3, 290–294. doi: 10.1002/epi4.12114, PMID: 29881810 PMC5983141

[ref35] Mejía-Granados D. M. Villasana-Salazar B. Lozano-García L. Cavalheiro E. A. Striano P. (2021). Gut-microbiota-directed strategies to treat epilepsy: clinical and experimental evidence. Seizure 90, 80–92. doi: 10.1016/j.seizure.2021.03.009 33762166

[ref36] Mohamed Qadir R. Assafi M. S. (2021). The association between body mass index and the oral Firmicutes and Bacteroidetes profiles of healthy individuals. Malays. Fam. Physician. 16, 36–43. doi: 10.51866/oa1129 34938391 PMC8680938

[ref37] Newman L. (2000). AHRQ's evidence-based practice centres prove viable. Agency for Healthcare Research and Quality. Lancet 356:1990. doi: 10.1016/s0140-6736(05)72963-9 11130533

[ref38] Olson C. A. Vuong H. E. Yano J. M. Liang Q. Y. Nusbaum D. J. Hsiao E. Y. (2018). The gut microbiota mediates the anti-seizure effects of the ketogenic diet. Cell 174:497. doi: 10.1016/j.cell.2018.06.051, PMID: 30007420 PMC6062008

[ref39] Page M. J. McKenzie J. E. Bossuyt P. M. . (2021). The PRISMA 2020 statement: an updated guideline for reporting systematic reviews. BMJ 372:n71. doi: 10.1136/bmj.n71, PMID: 33782057 PMC8005924

[ref40] Peng A. Qiu X. Lai W. Li W. Zhang L. Zhu X. . (2018). Altered composition of the gut microbiome in patients with drug-resistant epilepsy. Epilepsy Res. 147, 102–107. doi: 10.1016/j.eplepsyres.2018.09.013 30291996

[ref41] Rajilić-Stojanović M. Shanahan F. Guarner F. de Vos W. M. (2013). Phylogenetic analysis of dysbiosis in ulcerative colitis during remission. Inflamm. Bowel Dis. 19, 481–488. doi: 10.1097/MIB.0b013e31827fec6d 23385241

[ref42] Reddy S. D. Clossen B. L. Reddy D. S. (2018). Epigenetic histone deacetylation inhibition prevents the development and persistence of temporal lobe epilepsy. J. Pharmacol. Exp. Ther. 364, 97–109. doi: 10.1124/jpet.117.244939, PMID: 29101217

[ref43] Rodenhouse A. Talukder M. A. H. Lee J. I. Govindappa P. K. O’Brien M. Manto K. M. . (2022). Altered gut microbiota composition with antibiotic treatment impairs functional recovery after traumatic peripheral nerve crush injury in mice: effects of probiotics with butyrate producing bacteria. BMC. Res. Notes 15:80. doi: 10.1186/s13104-022-05967-8 35197129 PMC8867741

[ref44] Scheffer I. E. Berkovic S. Capovilla G. Connolly M. B. French J. Guilhoto L. . (2017). ILAE classification of the epilepsies: position paper of the ILAE Commission for Classification and Terminology. Epilepsia 58, 512–521. doi: 10.1111/epi.13709, PMID: 28276062 PMC5386840

[ref45] Schloissnig S. Arumugam M. Sunagawa S. Mitreva M. Tap J. Zhu A. . (2013). Genomic variation landscape of the human gut microbiome. Nature 493, 45–50. doi: 10.1038/nature11711, PMID: 23222524 PMC3536929

[ref46] Sutter R. Rüegg S. Tschudin-Sutter S. (2015). Seizures as adverse events of antibiotic drugs: a systematic review. Neurology 85, 1332–1341. doi: 10.1212/WNL.0000000000002023 26400582

[ref47] Thijs R. D. Surges R. O'Brien T. J. Sander J. W. (2019). Epilepsy in adults. Lancet 393, 689–701. doi: 10.1016/S0140-6736(18)32596-0 30686584

[ref48] Tremlett H. Bauer K. C. Appel-Cresswell S. Finlay B. B. Waubant E. (2017). The gut microbiome in human neurological disease: a review. Ann. Neurol. 81, 369–382. doi: 10.1002/ana.24901 28220542

[ref49] Türay S. Cangür Ş. Kahraman G. Kayabaşı E. Çetiner Ö. F. Aydın B. . (2023). Can the gut microbiota serve as a guide to the diagnosis and treatment of childhood epilepsy? Pediatr. Neurol. 145, 11–21. doi: 10.1016/j.pediatrneurol.2023.04.006, PMID: 37245274

[ref50] Ułamek-Kozioł M. Czuczwar S. J. Januszewski S. Pluta R. (2019). Ketogenic diet and epilepsy. Nutrients 11:2510. doi: 10.3390/nu11102510, PMID: 31635247 PMC6836058

[ref51] Wan L. Yang G. Zhang S. Sun Y. Li Z. Wang J. . (2021). Investigation of the association between imbalance of the intestinal flora and infantile spasms: a pilot case-control study. Transl. Pediatr. 10, 819–833. doi: 10.21037/tp-20-384, PMID: 34012831 PMC8107841

[ref52] Weis A. M. Round J. L. (2021). Microbiota-antibody interactions that regulate gut homeostasis. Cell Host Microbe 29, 334–346. doi: 10.1016/j.chom.2021.02.009, PMID: 33705705 PMC7990058

[ref53] World Health Organization. Epilepsy [fact sheet]. (2024-02-07) (2024). Available at: https://www.who.int/news-room/fact-sheets/detail/epilepsy (Accessed October 18, 2024).

[ref54] Xie G. Zhou Q. Qiu C. Z. Dai W. K. Wang H. P. Li Y. H. . (2017). Ketogenic diet poses a significant effect on imbalanced gut microbiota in infants with refractory epilepsy. World J. Gastroenterol. 23, 6164–6171. doi: 10.3748/wjg.v23.i33.6164, PMID: 28970732 PMC5597508

[ref55] Xu L. Chen D. Zhao C. Jiang L. Mao S. Song C. . (2021). Decreased abundance of Akkermansia after adrenocorticotropic hormone therapy in patients with West syndrome. BMC Microbiol. 21:126. doi: 10.1186/s12866-021-02189-z, PMID: 33892634 PMC8063292

[ref56] Yan Y. Li K. Jiang J. Jiang L. Ma X. Ai F. . (2023). Perinatal tissue-derived exosomes ameliorate colitis in mice by regulating the Foxp 3 + Treg cells and gut microbiota. Stem Cell Res Ther 14:43. Published 2023 Mar 20. doi: 10.1186/s13287-023-03263-1, PMID: 36941715 PMC10029206

[ref57] Younus I. Reddy D. S. (2017). Epigenetic interventions for epileptogenesis: a new frontier for curing epilepsy. Pharmacol. Ther. 177, 108–122. doi: 10.1016/j.pharmthera.2017.03.002, PMID: 28279785 PMC5565684

[ref58] Zeng Y. Cao S. Yang H. (2023). Roles of gut microbiome in epilepsy risk: a Mendelian randomization study. Front. Microbiol. 14:1115014. doi: 10.3389/fmicb.2023.1115014, PMID: 36922970 PMC10010438

[ref59] Zhai Q. Feng S. Arjan N. Chen W. (2019). A next generation probiotic, *Akkermansia muciniphila*. Crit. Rev. Food Sci. Nutr. 59, 3227–3236. doi: 10.1080/10408398.2018.1517725 30373382

[ref60] Zhang Z. Cheng N. Liang J. Deng Y. Xiang P. Hei Z. . (2023). Gut microbiota changes in animal models of spinal cord injury: a preclinical systematic review and meta-analysis. Ann. Med. 55:2269379. doi: 10.1080/07853890.2023.2269379, PMID: 37851840 PMC10586076

[ref61] Zhou C. Gong S. Xiang S. Liang L. Hu X. Huang R. . (2022). Changes and significance of gut microbiota in children with focal epilepsy before and after treatment. Front. Cell. Infect. Microbiol. 12:965471. doi: 10.3389/fcimb.2022.965471, PMID: 36405958 PMC9671114

