# Supplementary figures and images for: Changes in gut flora in patients with epilepsy: a systematic review and meta-analysis
Source: Front Microbiol. 2024 Nov 14;15:1480022. doi: 10.3389/fmicb.2024.1480022 (PMC11602489; doi:10.3389/fmicb.2024.1480022)

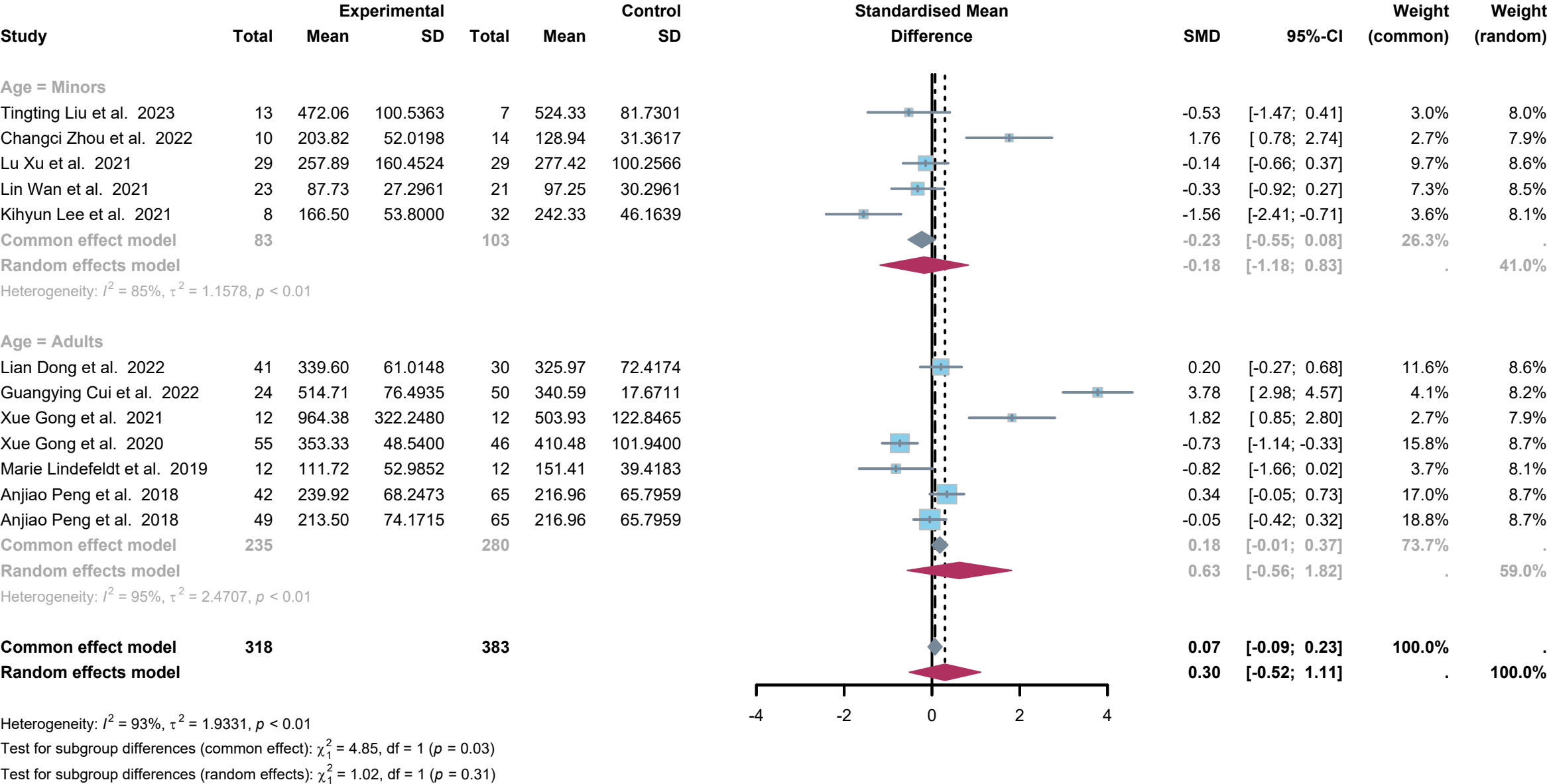

Supplement: Supplementary file 1 [file Image_1.pdf]

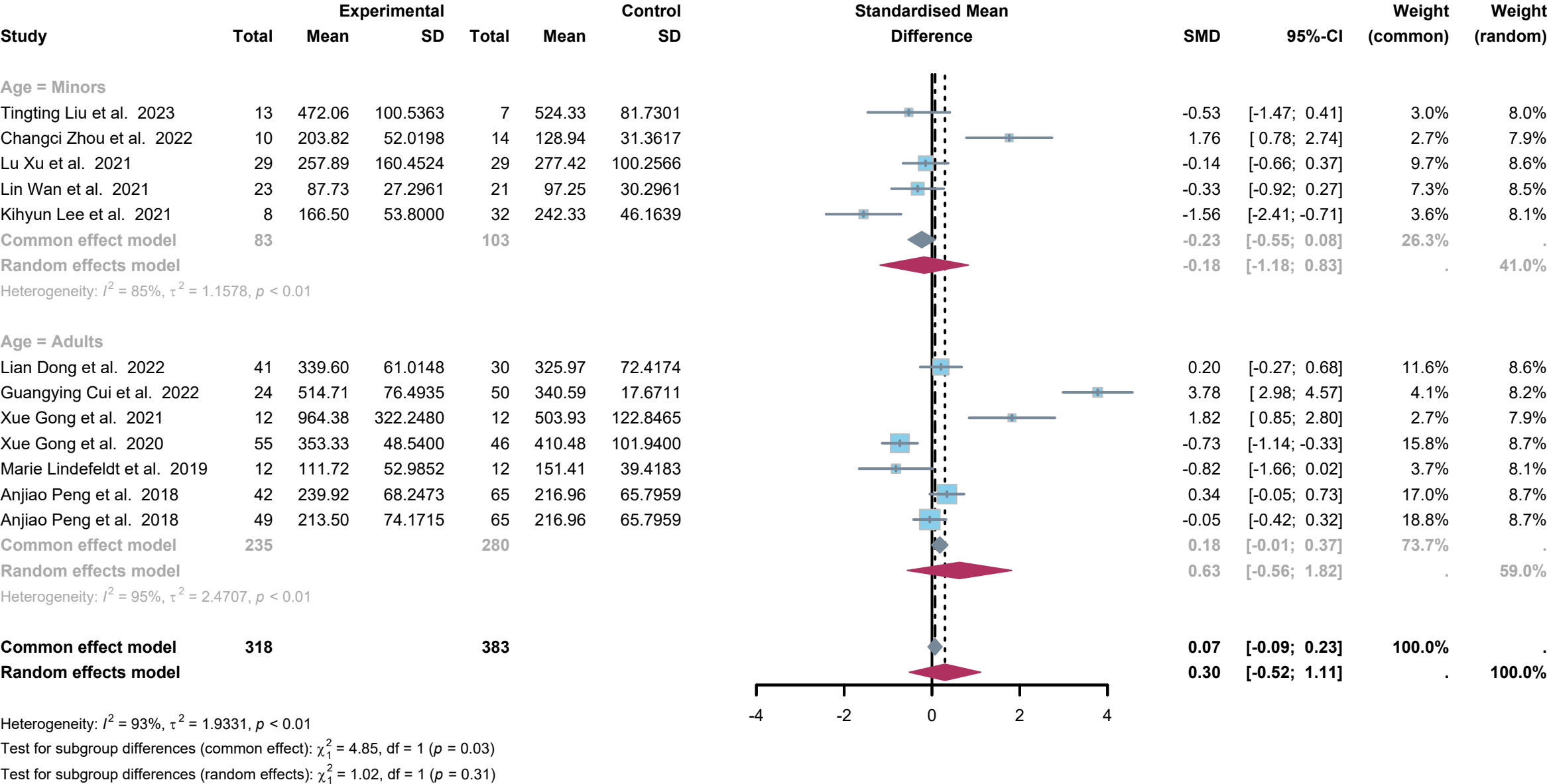

Supplement: Supplementary file 2 [file Image_2.pdf]

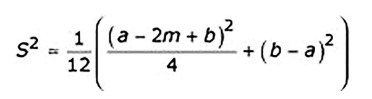

Supplement: Supplementary file 3 [file Image_3.jpg]
