# Supplementary material for: Changes in gut flora in patients with epilepsy: a systematic review and meta-analysis
Source: Front Microbiol. 2024 Nov 14;15:1480022. doi: 10.3389/fmicb.2024.1480022 (PMC11602489; doi:10.3389/fmicb.2024.1480022)
Supplement: Supplementary file 4 [file Presentation_1.pdf]

1. **Chao1 index**

   It is a non-parametric estimator of the number of unobserved species in a sample. The Chao1 index estimates total species richness based on the number of species that occur only once (singletons) and twice (doubletons) in the sample[1]. The formula for calculating the Chao1 index is as follows:

   $$S_{chao1} = S_{obs} + \frac{\mathrm{F1}^2}{2\mathrm{F2}}$$

   In this context, the variable "Sobs" represents the number of species observed, "F1" denotes the number of species observed on a single occasion, and "F2" signifies the number of species observed on two occasions.

2. **Shannon index**

   The Shannon index is a diversity index based on information theory that takes into account both species richness and evenness[2]. The index is calculated using the following formula:

   $$H' = -\sum(p_i \times \ln(p_i))$$

   The term "pi" represents the number of individuals belonging to the ith species, expressed as a proportion of the total number of individuals.

3. **Simpson index**

   The Simpson index is another frequently employed diversity index, which gauges the uniformity of species within a community. The calculation of the Simpson index is as follows:

   $$D = \sum(n_i^2/N^2)$$

   The value of Simpson's index is calculated by dividing the number of individuals of the ith species, represented by the variable ni, by the total number of individuals, represented by the variable N. A value of 1 indicates that one or a few species occupy the majority of individuals, which is indicative of low diversity within the community.

[1] Chao, A. (1984). Non-parametric estimation of the number of classes in a population. Scandinavian Journal of Statistics, 11, 265-270.

[2] Shannon CE. The mathematical theory of communication. 1963. *MD Comput*. 1997;14(4):306-317.

[3] Simpson, E. H. (1949). Measurement of diversity. Nature, 163, 688.
